# Comprehensive Metabolomic Profiling of Skin Lesions from Psoriasis Patients Reveals Disease Signatures

**DOI:** 10.7150/ijbs.134115

**Published:** 2026-05-29

**Authors:** Peiyao Zhu, Yuanyuan Wang, Jie Sun, Jiangluyi Cai, Zeyu Chen, Lian Cui, Xinyi Song, Yuye Wang, Zengyang Yu, Yu Gong, Qian Yu, Yuling Shi

**Affiliations:** 1 Department of Dermatology, Shanghai Skin Disease Hospital, Tongji University School of Medicine, 1278 Baode Road, Jing'an District, Shanghai 200443, China.; 2 Institute of Psoriasis, Tongji University School of Medicine, Shanghai, China.; 3 Department of Dermatology, Shanghai Tenth People's Hospital, Tongji University School of Medicine, 301 Yanchang Road, Jing'an District, Shanghai 200072, China.

**Keywords:** psoriasis, metabolomics, keratinocytes, neutrophils

## Abstract

Psoriasis is a chronic skin disease caused by dysregulated immune system. Although inflammation plays a vital role, the precise chemical changes within the skin remain unclear and metabolomic profiling of skin lesions has been limited by small sample sizes and narrow metabolite coverage. In this study, we performed wide-targeted metabolomics of skin samples from 30 psoriasis patients and 30 healthy controls to identify differential metabolites that may drive the disease. We identified 707 differential metabolites across 21 classes, including amino acids, fatty acids, organic acids, nucleotides, and others. Among them, two metabolites, UDP-N-acetyl-3-O-(1-carboxyvinyl)-D-glucosamine and ethylparaben, were strongly linked to psoriasis. Further analysis revealed that these changes were driven by specific differentiated keratinocytes and involved metabolomic enzyme-encoding genes related to nucleotide and pyrimidine metabolism pathways. To identify metabolites associated with disease severity, we grouped patients by PASI and BSA scores and identified 27 metabolites that increased as the disease worsened via Mfuzz clustering analysis. Among them, citrate and L-tyrosine significantly exacerbated disease by increasing skin thickness and inflammation after validation in vivo. Our findings provide a comprehensive map of metabolic changes in psoriatic skin and highlight citrate and L-tyrosine as potential indicators for disease severity and promising targets for future treatments.

## Introduction

Psoriasis is a chronic, immune-mediated inflammatory skin disease clinically characterized by erythematous papules and plaques covered with silvery scales. Its pathogenesis is complex and not yet fully understood, involving genetic susceptibility, environmental triggers, and dysregulated immune responses 1. The IL-23/IL-17A axis is considered a central pathogenic pathway, accompanied by elevated levels of pro-inflammatory cytokines such as IL-17A, IL-1β, TNF-α, IFN-γ, CXCL1, CXCL2, and CXCL8 2-5. Beyond cutaneous manifestations, psoriasis is associated with various systemic comorbidities, including cardiovascular disease, metabolic disorders, hepatorenal dysfunction, autoimmune conditions, and psychological disturbances 6, 7. Notably, the high prevalence of metabolism-related abnormalities such as obesity, diabetes, dyslipidemia, and metabolic syndrome, suggested that metabolic dysregulation may play a fundamental role in psoriasis progression and correlate with disease severity 8-11. Recent findings from our group indicated that decreased PPARα expression could downregulate FADS2, thereby impairing DHA synthesis within fatty acid metabolism and consequently exacerbating psoriatic inflammation 12. Itaconate (ITA) and its derivatives, including 4-octyl itaconate (4-OI) and dimethyl itaconate (DMI), could ameliorate imiquimod (IMQ) -induced psoriasis by suppressing Th17 cells frequency via regulating mitophagy 13. Elevated adenosine levels primarily generated from a population of CD73^high^ fibroblasts in psoriatic skin closely correlated with disease severity and through ADORA2B, adenosine could mediate communication between fibroblasts and keratinocytes (KCs), causing mitochondrial dysfunction and oxidative stress that ultimately resulted in the release of pro-inflammatory mediators from KCs 14. Meanwhile, gut microbe-derived metabolites like indoxyl sulfate or histamine could also drive psoriatic inflammation by modulation of skin Th17 cells chromatin accessibility or promoting γδT17 cell differentiation in an Hrh1 receptor-dependent manner 15, 16.

With the rapid advancement of targeted and untargeted metabolomics, numerous differential metabolites have been identified in psoriasis, primarily at the plasma or serum level, as blood samples are more readily accessible than skin lesions 17, 18. Lipidomics profiling revealed significant alterations in glycerophospholipid metabolism, with increased levels of lysophosphatidic acid (LPA), lysophosphatidylcholine (LysoPC), and phosphatidic acid (PA), alongside decreased phosphatidylinositol (PI) and phosphatidylcholine (PC) in psoriasis patients 19. A large-scale plasma metabolome analysis further identified elevated ethanolamine phosphate and reduced levels of XA0019, nicotinic acid, and 20α-hydroxyprogesterone in psoriatic individuals 20. In addition, targeted metabolomic profiling of amino acids and carnitines in both patient sera and IMQ-induced psoriasis-like mice revealed significant alterations in essential amino acids, branched-chain amino acids, and L-carnitine (C0) associated with disease pathogenesis 17.

However, metabolomic studies focusing on skin lesions or biopsies from psoriasis patients remain limited to small sample sizes and single-class metabolite coverage 21, 22. For instance, a full-spectrum metabolomic analysis of lesional skin from 20 plaque psoriasis patients versus 19 healthy controls identified only 29 significantly altered metabolites, primarily characterized by features of increased cell proliferation 23. Similarly, lipidomic profiling of skin lesions from IMQ-induced mice and psoriasis patients showed elevated levels of ceramide phosphates (CerP) and ceramides, further stratifying patients into CC1 and CC2 subtypes based on higher ceramide-1-phosphate (C1P) and uric acid levels 24. These findings underscore the need for comprehensive, large-scale metabolomic profiling of psoriatic lesions to fully elucidate disease-related metabolic dysregulation.

A growing body of evidence suggested that metabolic alterations in psoriatic skin lesions or plasma were closely associated with the therapeutic efficacy of conventional treatments and biologics 25-27. However, relapse at previously affected sites following treatment discontinuation remains a critical challenge, even in the era of achieving Psoriasis Area and Severity Index (PASI) 90 or 100 responses 28. Emerging evidence suggests that both immune cells, such as tissue-resident memory T (T_RM_) cells, Langerhans cells (LCs), dermal dendritic cells (dDCs), and non-immune cells, including epithelial stem cells and fibroblasts, could retain inflammatory memory within resolved lesions, thereby potentially contributing to disease recurrence 29. These cellular populations were modulated by local immune microenvironmental factors within the lesional skin. Although IL-23 blockade significantly suppressed inflammatory gene expression in KCs, its effect on T_RM_ cells in the lesional skin remained unclear. Moreover, despite long-term TNF-α inhibitor therapy substantially alleviating clinical symptoms, T cells in healed lesions continued to express IL-17 30. Concurrently, previous studies have demonstrated that highly proliferative basal KCs in psoriasis outcompete other cells for glucose uptake, leading to impaired KC differentiation 31, 32. Collectively, these findings underscore the need for comprehensive metabolomic profiling of psoriatic lesions to better understand disease progression and identify mechanisms underlying relapse.

In this study, we performed an integrated, broad-targeted metabolomic analysis based on LC-MS/MS on skin lesions from 30 patients with psoriasis vulgaris and 30 healthy controls, aiming to characterize psoriatic metabolic signatures associated with disease severity 33. A total of 707 differentially abundant metabolites were identified, encompassing diverse classes including alcohol and amines, amino acid and its metabolites, benzene and substituted derivatives, carbohydrate and its metabolites, fatty acid, organic acid and its derivatives, and nucleotide and its metabolites. Correlation analysis with clinical severity indices, PASI and Body Surface Area (BSA) scores, were conducted to identify metabolites linked to disease severity. Notably, differential nucleotide and its metabolites were significantly enriched in nucleotide metabolism and pyrimidine metabolism pathways. Through integrative analysis with public bulk RNA-seq 34 and single-cell RNA-seq (scRNA-seq) 35 datasets from psoriatic lesions, we identified key genes and specific cell types potentially responsible for the production or regulation of these metabolites. Finally, three candidate metabolites (citrate, L-tyrosine, and purine), which positively correlated with PASI and BSA scores, were validated in an IMQ-induced psoriasis-like dermatitis mouse model. In summary, this study provided a comprehensive metabolomic profiling of psoriatic lesions across nearly all metabolite classes and revealed potential metabolic signatures associated with disease severity in psoriasis.

## Materials and Methods

### Collection of patient samples

Skin lesion samples were collected via biopsy from patients with psoriasis vulgaris enrolled in the Shanghai Psoriasis Effectiveness Evaluation CoHort (SPEECH), a clinical trial registered under the Chinese Clinical Trial Registry (ChiCTR2000036186). The study protocol was approved by the Ethics Committee of Shanghai Skin Disease Hospital (Approval No. 2024-08). Inclusion criteria were age ≥18 years, a diagnosis of chronic moderate-to-severe plaque psoriasis, and no systemic treatment, phototherapy, or biologics within the previous three months. Normal skin samples were obtained from healthy controls as comparators. All specimens were immediately snap-frozen in liquid nitrogen after excision to prevent repeated freeze-thaw cycles. PASI and BSA scores were thoroughly evaluated and recorded for all enrolled patients.

### Procedure of TM wide-targeted metabolomics

Skin lesion samples from psoriasis patients and healthy controls were prepared following standard procedures for TM broad-targeted metabolomics (Metware Biotechnology, China). Supernatant aliquots were pooled to create quality control (QC) samples and analyzed by LC-QTOF-MS/MS for non-targeted detection. Metabolite identification was performed using the self-built MWDB database (including secondary spectra and retention time), integrated public databases (Metlin, HMDB, KEGG), AI prediction library, and MetDNA. After identification, multiple reaction monitoring (MRM) transitions were extracted and combined with Metware's target database to construct a project-specific library. All samples underwent MRM-based accurate quantification on a QTRAP platform.

Three LC/MS methods were applied: (1) positive ion mode with T3 column (0.1% formic acid in water/acetonitrile gradient); (2) negative ion mode with T3 column (same gradient); (3) negative ion mode with HILIC column (ammonium formate buffer gradient). The UPLC system (ExionLC AD) coupled with QTRAP MS/MS (Sciex) operated under optimized source parameters: temperature 550°C, ion spray voltage 5500V (positive)/-4500V (negative), with gas settings of 50/50/25 psi. TOF MS scans covered m/z 50-1000, and product ion scans m/z 25-1000. Data acquisition was controlled by Analyst 1.6.3 software, with MRM transitions monitored according to metabolite elution periods.

### Identification of differential metabolites

To identify differentially abundant metabolites, we applied the Orthogonal Partial Least Squares-Discriminant Analysis (OPLS-DA) model, which allows discrimination between groups by maximizing class separation. The variable importance in projection (VIP) score was used to rank metabolites based on their contribution to group discrimination, with metabolites having VIP > 1 considered as significantly contributing to the separation. In parallel, univariate statistical analysis was performed using Student's t-test to assess the statistical significance of individual metabolite differences between groups. Differential metabolites were defined based on the following combined criteria: (1) VIP > 1, reflecting a substantial contribution to intergroup discrimination in the multivariate model; and (2) P-value < 0.05, indicating statistically significant differences between groups. This dual-filtering strategy integrates both multivariate and univariate approaches to ensure robust and reliable identification of metabolites that are genuinely different between conditions.

### Enrichment analysis of differential metabolites

Identified metabolites were annotated using the KEGG Compound database, and the annotated metabolites were subsequently mapped to KEGG Pathway databases to identify relevant metabolic pathways. Metabolite sets enrichment analysis (MSEA) was then performed on pathways containing significantly regulated metabolites, with statistical significance determined by the hypergeometric test (P-value < 0.05). This approach enabled the identification of key metabolic pathways potentially involved in the pathogenesis of psoriasis.

### Bulk and scRNA-seq data analysis

Bulk RNA-seq data (GSE13355) from psoriatic and normal skin were downloaded from the GEO database 34, and scRNA-seq data from psoriatic lesions and healthy controls were obtained from a previously published study 35. KEGG enrichment analysis was performed on the GSE13355 dataset to identify differentially expressed genes involved in metabolic pathways that overlapped with those enriched by our differential metabolite set. The scRNA-seq data were then used to pinpoint the cellular origins of these metabolites by mapping key metabolic genes which were identified from the bulk RNA-seq analysis to specific cell types within psoriatic lesions. All analysis and visualizations were conducted using the Metware Cloud platform, a freely available online tool for data processing and graphing.

### Correlation analysis

Spearman correlation analysis was performed to assess the relationship between the relative expression levels of differential metabolites and clinical severity scores (PASI and BSA) in psoriasis patients and healthy controls. To further identify metabolites with expression patterns continuously and positively correlated with disease severity, Mfuzz clustering analysis based on fuzzy C-means algorithm was conducted using R (version 4.1.0) via the Metware Cloud platform. This approach enabled the grouping of metabolites exhibiting similar abundance trends associated with increasing PASI and BSA scores.

### Mice and treatment

Seven-week-old C57BL/6 mice were purchased from Shanghai Regen Biotechnology Co., Ltd. and housed under specific pathogen-free (SPF) conditions. All animal experiments were approved by the Ethics Committee of Shanghai Skin Disease Hospital (Approval No. 2024-20). To validate the metabolites positively correlated with disease severity in psoriasis, we established an IMQ-induced psoriasis-like dermatitis mouse model. The dorsal skin of each mouse was shaved (approximately 2.5 cm×2.5 cm area) and topically treated with 62.5 mg of 5% IMQ cream once daily for 4-5 consecutive days. Body weight and lesion photographs were recorded daily. For metabolite validation, mice were randomly assigned to experimental groups as described below and all intraperitoneal (i.p.) injections were performed under sodium pentobarbital anesthesia: (1) Citrate validation: Mice were divided into three groups: saline control, IMQ+vehicle, and IMQ+citrate. Citrate (Aladdin, China) was dissolved in 100μL saline and administered i.p. at 240 mg/kg daily for 4 consecutive days; (2) L-Tyrosine validation: Mice were assigned to IMQ+vehicle and IMQ+L-tyrosine groups. L-Tyrosine (MCE, USA) was dissolved in 150μL of vehicle (50% PEG300+50% saline) and injected i.p. at a final concentration of 2 mM daily for 4 days; (3) Purine validation: Mice were assigned to IMQ+vehicle and IMQ+purine groups. Purine (MCE, USA) was dissolved in 300μL of vehicle (10% DMSO+90% corn oil, Aladdin, China) and administered i.p. at 100 mg/kg every other day for 5 days. Control groups for each metabolite received an equal volume of the corresponding vehicle via i.p. injection. At the end of treatment, mice were euthanized by cervical dislocation. Dorsal skin samples were collected for: (1) hematoxylin and eosin (H&E) staining to measure epidermal thickness; (2) quantitative real-time PCR (qPCR) to assess inflammatory cytokine expression; and (3) flow cytometry to evaluate the proportions of immune cell subsets in lesional skin.

### Real time quantitative PCR (qPCR)

Skin samples from mice were extracted for the isolation of mRNA and synthesis of cDNA. Fluorescence qPCR was carried out with specific primer pairs in accordance with the detailed procedures as described 36.

### Multiplex immunofluorescence (IF)

Paraffin-embedded sections of skin lesions from psoriasis patients were subjected to gradient rehydration. Antigen retrieval was performed using EDTA antigen retrieval solution (pH=9.0, Beyotime, China). Multiplex staining was carried out using a commercial multiplex IF kit (Aifang Biological, China). Briefly, sections were treated with peroxidase blocking solution to quench endogenous peroxidase activity, followed by washing with PBS three times for 5 min each. Non-specific binding was blocked with normal goat serum for 30 min, after which sections were incubated with specific primary antibodies for 1-2 h at 37 °C in a humidified chamber protected from light. After washing, sections were incubated with Polymer-HRP secondary antibody for 30 min at room temperature in the dark, followed by washing. Tyramide signal amplification (TSA) fluorochrome was then applied for 5-10 min in the dark. Sections were washed again, and the antibody stripping step was performed by repeating the antigen retrieval procedure to enable subsequent rounds of multiplex labeling. Finally, nuclei were counterstained with DAPI (Beyotime, China), and sections were mounted for microscopic examination. The following specific primary antibodies were used: anti-UPP1 Rabbit Polyclonal Antibody, anti-TYMP Rabbit Polyclonal Antibody, anti-PNP Rabbit Polyclonal Antibody, and anti-AMPD3 Rabbit Polyclonal Antibody (all from Proteintech, China).

### Tissue processing and flow cytometry

Mouse skin samples were collected and placed on aluminum foil, and the subcutaneous tissue was carefully scraped off. The remaining skin tissue was weighed (approximately 50 mg) and then minced into fine pieces using surgical scissors. The minced tissue was transferred to a digestion solution (prepared fresh and mixed thoroughly to avoid clumping) and incubated at 37 °C with rotation for 1-1.5 h, with gentle shaking every 20 min to facilitate enzymatic dissociation. After digestion, 1 mL of fetal bovine serum (FBS) was added per tube to terminate the reaction, and the suspension was mixed gently. The digested mixture was passed through a 40 μm cell strainer into a 15 mL centrifuge tube. The original tube was rinsed with 9 mL of pre-cooled PBS, which was then filtered through the same strainer. The collected cells were centrifuged at 500 × g for 5 min at 4 °C. The supernatant was discarded, and the cell pellet was resuspended in 200 μL of staining buffer and transferred to a round-bottom 96-well plate. The plate was centrifuged at 500 × g for 5 min at 4 °C, and the supernatant was removed. Cells were resuspended in 100 μL of staining buffer and incubated with 2 μL of Fc Receptor blocker (Thermo Fisher, USA) for 10 min at room temperature in the dark. Subsequently, fluorochrome-conjugated antibodies were added (1 μL per well) and incubated at 4 °C for 30 min in the dark. After incubation, wells were filled with pre-cooled PBS, mixed gently, and centrifuged at 500 × g for 5 min at 4 °C. The supernatant was discarded, and cells were resuspended in 150 μL of staining buffer. The cell suspension was filtered through a cell strainer immediately before flow cytometry acquisition. Prior to analysis, cell viability dye was added to the staining buffer to a final volume of 400 μL. The following antibodies were used for immunophenotyping: Ms CD45 BV510 (Biolegend, USA), Ms CD3 Percp-Cy5.5 (Biolegend, USA), Ms Ly6G APC-Cy7 (Tonbo Bioscience, USA), Ms EpCAM AF700 (Biolegend, USA), Ms F4/80 PE (Biolegend, USA), Ms CD11c APC (Biolegend, USA), Ms CD11b AF488 (Biolegend, USA), and Sytox™ Blue (Thermo Fisher, USA) for viability staining.

### Statistical analysis

Unsupervised principal component analysis (PCA) was performed by statistics function prcomp within R software. The data was scaled to unit variance before unsupervised PCA. Enrichment of KEGG pathways was performed by R software with ggplot2 3.3.0. Combined radar chart was performed by R software with ggplot2 3.3.6. Graphs of upsetR analysis were produced with R software with UpSetR 1.4.0. Schematic illustrations were created using BioRender software under a valid academic license. Statistical significance between two groups was assessed using a two-tailed Student's t-test. For comparisons among three or more groups, one-way analysis of variance (ANOVA) was applied, followed by appropriate post hoc tests. All data were represented as mean ± standard deviation (SD). A P value < 0.05 was considered statistically significant.

## Results

### Comprehensive differential metabolites identified by wide-targeted metabolomics

**Figure 1:**
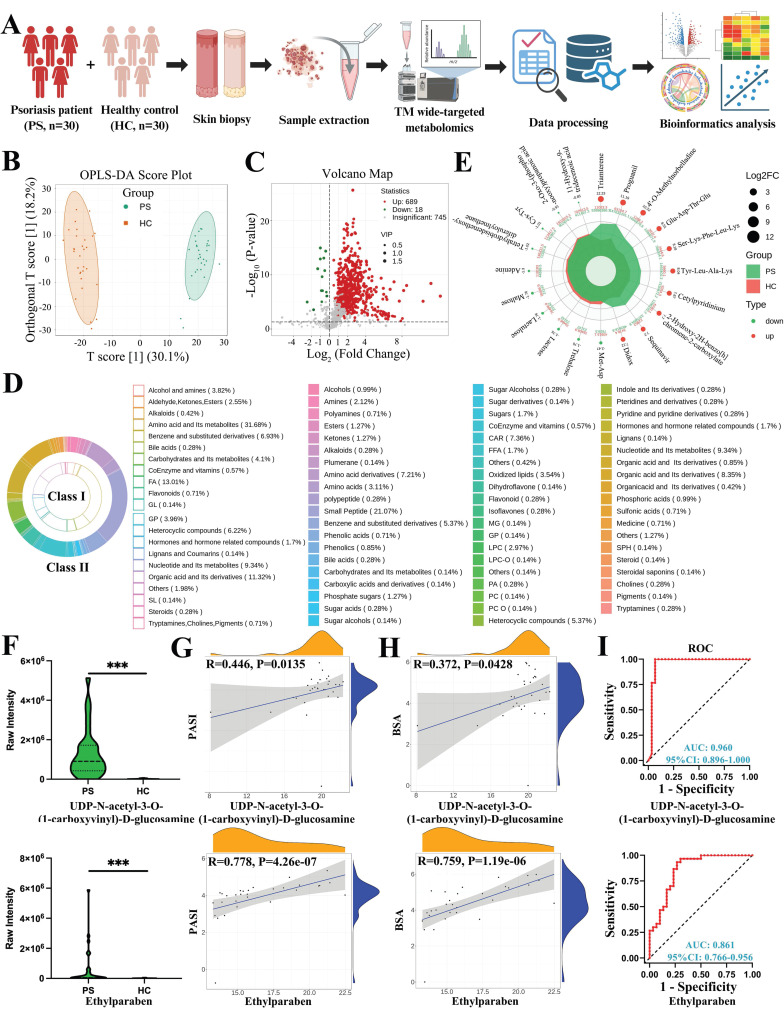
** Comprehensive differential metabolites identified by wide-targeted metabolomics and correlation analysis.** (A) Workflow of the study. (B) OPLS-DA score plot distinguishing psoriasis patients (PS) from healthy controls (HC) skin samples. (C) Volcano plot showing 689 up-regulated and 18 down-regulated metabolites in PS versus HC. (D) Classification of all differential metabolites into 21 Class I and 59 Class II categories. (E) Radar chart displaying the top 10 up-regulated and down-regulated metabolites. (F-I) Violin plots, correlation analyses with PASI and BSA scores, and receiver operating characteristic (ROC) curves for UDP-N-acetyl-3-O-(1-carboxyvinyl)-D-glucosamine and ethylparaben. FA, Fatty Acyls; GL, Glyceride; GP, Glycerophospholipid; SL, Sphingolipids; CAR, Acylcarnitine; FFA, Free Fatty Acids; MG, Monoglyceride; LPC, Lysophosphatidylcholine; LPC-O, O-alkyl-lysophosphatidylcholine; PA, Phosphatidic Acid; PC, Phosphatidylcholine; PC O, O-alkyl-phosphatidylcholine; SPH, Sphingosine.

In this study, a total of 60 skin samples were collected and divided into two groups, psoriasis (PS) and healthy control (HC), for metabolomic analysis. The overall analytical workflow was illustrated in Figure 1A, and the clinical characteristics of the enrolled participants were summarized in Table 1. Mixed samples were first analyzed using non-targeted metabolomics technology to broadly profile metabolite composition. Based on these data, a patented wide-targeted metabolomics approach combined with a self-built database was employed for further metabolic characterization, leading to the detection of 1452 metabolites. These metabolites were categorized into 24 major classes, including alcohols and amines, aldehydes, ketones, esters, amino acids and their metabolites, carbohydrates and their metabolites, among others. OPLS-DA was applied to filter differential variables by eliminating group-irrelevant variation (Figure 1B). Volcano plot analysis revealed that, compared with healthy controls, psoriatic lesions contained 689 up-regulated and 18 down-regulated metabolites (Figure 1C). A scatter plot was generated to visualize differences in the relative abundance of various metabolite classes between the two groups (Figure S1A).

All differential metabolites were further classified into 21 Class I and 59 Class II categories. The top ten Class I categories, ranked by metabolite proportion, were as follows: amino acids and their metabolites (31.68%, Table S1); fatty acids (FAs, 13.01%, Table S2); organic acids and derivatives (11.31%, Table S3); nucleotides and their metabolites (9.34%, Table S4); benzene and substituted derivatives (6.93%, Table S5); heterocyclic compounds (6.22%, Table S6); carbohydrates and their metabolites (4.10%, Table S7); glycerophospholipids (GPs, 3.96%, Table S8); alcohols and amines (3.82%, Table S9); and aldehydes, ketones, and esters (2.55%, Table S10). Additional metabolites were listed in Table S11. The Class II categories provided finer resolution of metabolite subtypes (Figure 1D). A heat map displaying all differential metabolites between PS and HC groups is presented in Figure S1B.

The top ten up-regulated and down-regulated metabolites, ranked by log₂(fold change, FC), were shown in the radar chart (Figure 1E). The most highly up-regulated metabolites included triamterene (heterocyclic compounds), proguanil (alcohols and amines), 4'-O-methylnorbelladine (alkaloids), Glu-Asp-Thr-Glu, Ser-Lys-Phe-Leu-Lys, Tyr-Leu-Ala-Lys (amino acid metabolites, small peptides), cetylpyridinium (heterocyclic compounds), 2-hydroxy-2H-benzo[h]chromene-2-carboxylate (organic acid derivatives), saquinavir (others), and didox (benzene and substituted derivatives). The most down-regulated metabolites included Met-Asp (amino acid metabolites, small peptides), maltose, lactulose, lactose, trehalose (carbohydrates and their metabolites), adenine (nucleotides and their metabolites), tetrahydrobisdemethoxydiferuloylmethane (aldehydes, ketones, esters), Cys-Tyr (amino acid metabolites, small peptides), 2-oxo-3-(phosphonooxy)propanoic acid (organic acid derivatives), and 11-hydroxy-9-tridecenoic acid (fatty acids). Endogenous and exogenous differential metabolites were annotated accordingly. Given the inclusion of numerous small peptides with limited functional relevance to cellular biology and metabolism, we re-analyzed the top ten endogenous and exogenous differential metabolites after excluding small peptides. These results are presented in a heat map with corresponding log₂FC and P values (Figure S1C). For completeness, the top 20 differential metabolites among small peptides are listed in Table S12.

### Correlation analysis of endogenous and exogenous differential metabolites with PASI and BSA scores

Endogenous metabolites refer to substances naturally present in the body or generated during metabolic processes, including proteins, vitamins, and minerals. In contrast, exogenous metabolites are derived from external sources, such as dietary components (e.g., proteins, vitamins, minerals) or pharmacological agents (e.g., antibiotics, glucocorticoids). In this study, we analyzed the correlation between differential metabolites and clinical severity scores (PASI and BSA) to identify potential metabolic signatures associated with disease severity.

The top ten endogenous differential metabolites included triamterene (heterocyclic compounds), (2-hydroxy-2-oxo-1,2lambda5-oxaphospholan-5-yl)methyl (Z)-octadec-9-enoate (FA), UDP-N-acetyl-3-O-(1-carboxyvinyl)-D-glucosamine (nucleotide metabolites), methylcarbamyl PAF (GP), 1-oleoyl lysophosphatidic acid sodium salt (GP), orotidylic acid (nucleotide metabolites), uridine-5'-diphospho-N-acetylgalactosamine disodium salt (nucleotide metabolites), ethylparaben (benzene and substituted derivatives), carnitine C20:4 (FA), and 2,22-dideoxy-3-dehydroecdysone (hormones and related compounds). The top ten exogenous differential metabolites included proguanil (alcohols and amines), 4'-O-methylnorbelladine (alkaloids), cetylpyridinium (heterocyclic compounds), 2-hydroxy-2H-benzo[h]chromene-2-carboxylate (organic acid derivatives), saquinavir (others), didox (benzene and substituted derivatives), narirutin (flavonoids), pyrazolynate (benzene and substituted derivatives), H-Lys-Tyr-Lys-OH acetate salt (benzene and substituted derivatives), and 3-chloroaniline (benzene and substituted derivatives). Correlation analyses among these metabolites are presented in Figure S1D.

To evaluate the association between metabolite levels and disease severity, we performed correlation analyses with PASI and BSA scores. Among the endogenous metabolites, UDP-N-acetyl-3-O-(1-carboxyvinyl)-D-glucosamine and ethylparaben showed significant positive correlations with both PASI (Figure 1F and G) and BSA scores (Figure 1H). Receiver operating characteristic (ROC) curve analysis further demonstrated that both metabolites exhibited high area under the curve (AUC) values, supporting their potential as reliable diagnostic indicators for psoriasis (Figure 1I). Although other endogenous metabolites did not show consistent correlations with clinical scores, ROC analysis suggested that some may still hold diagnostic value (Figure S2).

In contrast, all ten exogenous metabolites exhibited inconsistent correlations with PASI and BSA scores, precluding their use as reliable indicators for disease severity assessment or accurate diagnosis (Figure S3). These findings highlight the importance of distinguishing between endogenous and exogenous metabolic alterations in the context of psoriasis and suggest that UDP-N-acetyl-3-O-(1-carboxyvinyl)-D-glucosamine and ethylparaben may serve as promising metabolic markers for disease evaluation.

### Nucleotide and pyrimidine metabolism among differential metabolites and expressed genes

**Figure 2:**
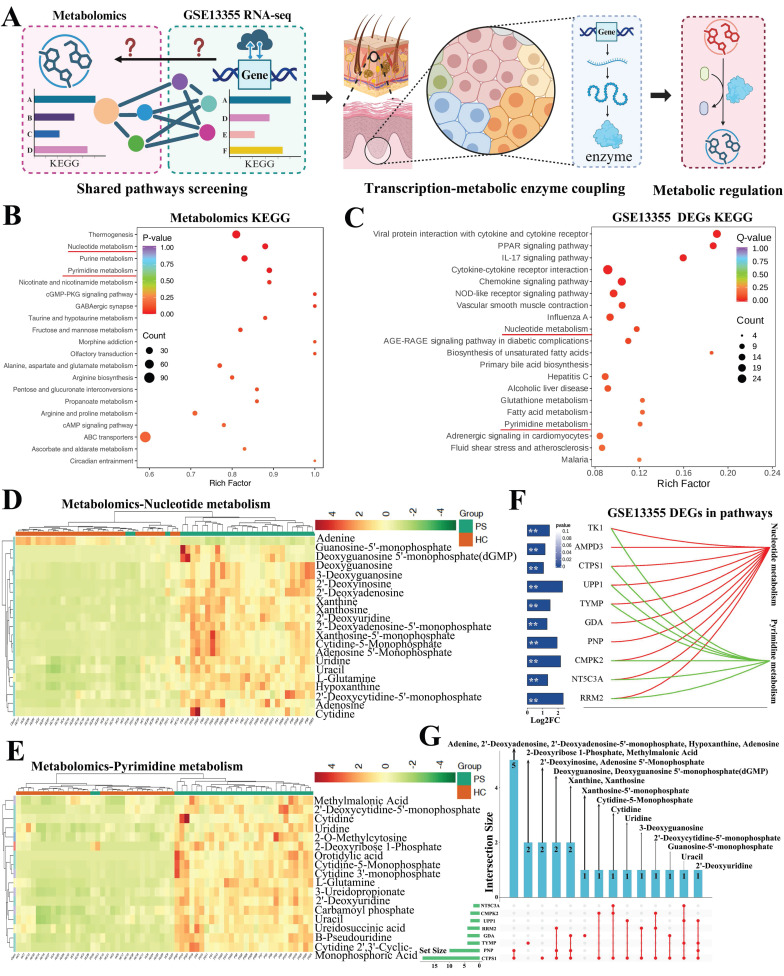
** Nucleotide and pyrimidine metabolism among differential metabolites and genes.** (A) Workflow for identifying shared metabolic pathways and genes encoding metabolic enzymes. (B) KEGG enrichment analysis of differential metabolites, showing the top 20 enriched pathways. (C) KEGG enrichment of differential genes from the GSE13355 dataset, highlighting nucleotide and pyrimidine metabolism as overlapping pathways. (D) Heat map of 21 differential metabolites involved in nucleotide metabolism. (E) Heat map of 17 differential metabolites involved in pyrimidine metabolism. (F) Differentially expressed genes within nucleotide and pyrimidine metabolism pathways, including *TK1*, *AMPD3*, *CTPS1*, *UPP1*, *TYMP*, *GDA*, *PNP*, *CMPK2*, *NT5C3A*, and *RRM2*. (G) UpSetR plot illustrating the regulatory relationships between genes and metabolites in both pathways.

KEGG pathway enrichment analysis was performed to elucidate the biological functions and metabolic pathways associated with the identified differential metabolites. This approach enables the identification of key biological processes in which these metabolites participate and provides insights into their potential impact on cellular physiology and disease phenotype. The analytical workflow was illustrated in Figure 2A. We first conducted KEGG enrichment analysis of all differential metabolites, which were categorized into six major classes, including organismal systems, metabolism, human diseases, genetic information processing, environmental information processing, and cellular processes (Figure S4A). The top 20 enriched metabolic pathways included thermogenesis, nucleotide metabolism, purine metabolism, pyrimidine metabolism, and nicotinate/nicotinamide metabolism, among others (Figure 2B).

Given the detection of numerous differential metabolites, we next sought to identify the genes potentially responsible for regulating the corresponding metabolic enzymes. To this end, we re-analyzed the publicly available bulk RNA-seq dataset GSE13355, which compares psoriatic lesions with normal skin. Differential expression analysis (|log₂FC| ≥ 1, *P* < 0.05) followed by KEGG enrichment revealed the top 20 metabolic pathways (Figure S4B and Figure 2C). Notably, both nucleotide metabolism and pyrimidine metabolism pathways overlapped between the metabolomics and transcriptomics datasets.

In the nucleotide metabolism pathway, a total of 21 differential metabolites were identified (Figure 2D). Similarly, the pyrimidine metabolism pathway contained 17 differential metabolites (Figure 2E). We next examined the differentially expressed genes within these two pathways, including *TK1*, *AMPD3*, *CTPS1*, *UPP1*, *TYMP*, *GDA*, *PNP*, *CMPK2*, *NT5C3A*, and *RRM2*, and their fold changes were summarized in Figure 2F. To further explore the regulatory relationships between genes and metabolites, we performed UpSetR analysis. The results indicated that metabolites such as adenine, 2'-deoxyadenosine, 2'-deoxyadenosine-5'-monophosphate, hypoxanthine, and adenosine may be regulated by *PNP* and *CTPS1* (Figure S4C). *CTPS1* was also implicated in the production of 2'-deoxyinosine and adenosine 5'-monophosphate. *TYMP* appeared to regulate methylmalonic acid and 2-deoxyribose 1-phosphate, while *UPP1* contributed to uridine production. Other genes, including *RRM2*, *NT5C3A*, *CTPS1*, and CMPK2, were associated with the production or conversion of the remaining metabolites (Figure S4D). An integrated regulatory network depicting these gene-metabolite interactions was presented in Figure 2G.

### Differentiated KCs contributed to abnormal nucleotide and pyrimidine metabolism

**Figure 3:**
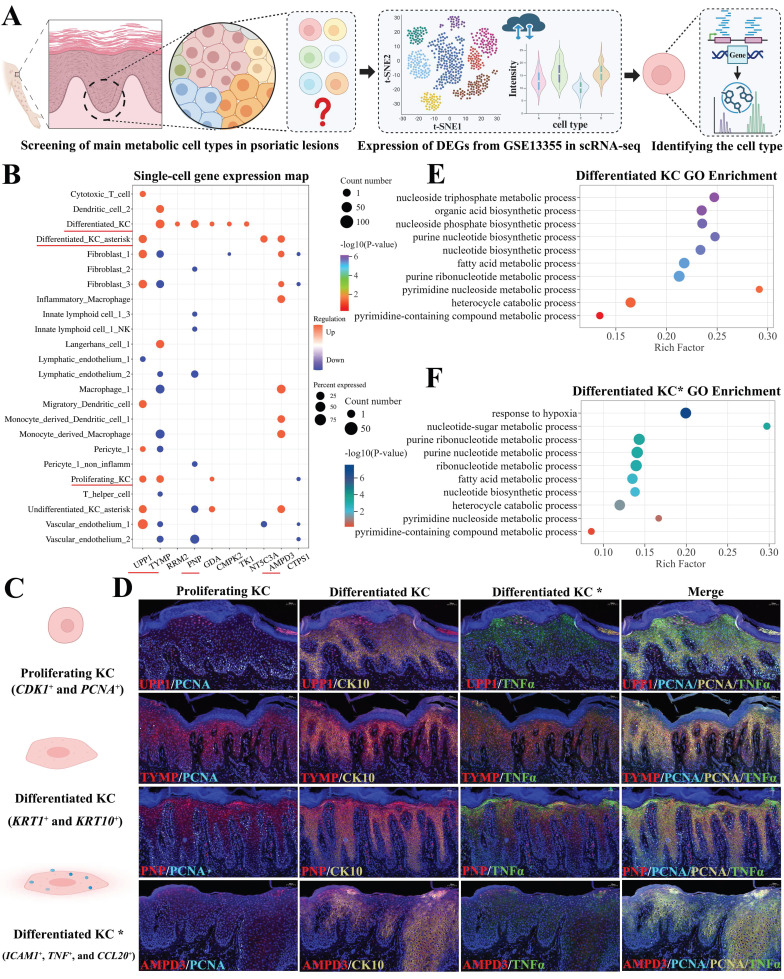
** Differentiated KCs contributed to abnormal nucleotide and pyrimidine metabolism.** (A) Workflow for identifying key metabolic cell types in psoriatic lesions. (B) ScRNA-seq analysis revealing 24 potential cell types, with differentiated KCs and their inflammatory subset (KC*) identified as primary mediators of metabolite production or conversion. (C) Marker genes defining proliferating KCs, differentiated KCs, and inflammatory differentiated KCs (KC*). (D) Multiplex IF staining of regulatory enzymes UPP1, TYMP, PNP, and AMPD4 in psoriatic lesions. (E-F) Gene Ontology (GO) enrichment analysis of differentiated KCs and inflammatory KC* from scRNA-seq data, showing enrichment of biological processes related to nucleotide and pyrimidine metabolism.

To identify the specific cell types responsible for regulating metabolic enzymes involved in nucleotide and pyrimidine metabolism, we re-analyzed publicly available scRNA-seq data from psoriatic lesions and normal skin. Cell types were annotated based on the expression levels of differentially expressed genes (|log₂FC| ≥ 1, P < 0.05) derived from the GSE13355 bulk RNA-seq dataset (Figure 3A). A total of 24 cell types were identified, including cytotoxic T cells, DC subsets, differentiated KCs, differentiated KCs in an inflammatory state (termed differentiated KC asterisk), and fibroblasts (subtypes 1-3), among others (Figure 3B). In light of the heterogeneous expression patterns of differentially expressed genes across cell types, we provisionally assigned differentiated KCs and differentiated KC asterisk as the main cell types responsible for metabolite production or conversion, while proliferating KCs and undifferentiated KC asterisk were considered to contribute to a lesser extent.

To further validate the role of differentiated KC subsets in regulating metabolic enzymes within nucleotide and pyrimidine metabolism pathways, we performed multiplex IF staining on psoriatic skin lesions using cell-type-specific markers derived from scRNA-seq data. Proliferating KCs were identified by *CDK1* and *PCNA* co-expression, differentiated KCs by *KRT1* and *KRT10*, and differentiated KC asterisk (inflammatory state) by *ICAM1*, *TNF*, and *CCL20* (Figure 3C). Using this approach, we examined the protein expression of key metabolic enzyme-encoding genes, including *UPP1*, *TYMP*, *PNP*, and *AMPD3*. UPP1 was predominantly expressed in differentiated KC asterisk. TYMP localized mainly to proliferating and differentiated KCs, with sparse expression in differentiated KC asterisk. PNP was expressed in both differentiated KCs and differentiated KC asterisk, while AMPD3 was primarily detected in differentiated KC asterisk, with weak expression in differentiated KCs (Figure 3D).

To functionally confirm whether differentiated KCs and their inflammatory subset regulate nucleotide and pyrimidine metabolism, we performed Gene Ontology (GO) enrichment analysis on the differentially expressed genes specific to these two cell types. In differentiated KCs, enriched biological processes included purine nucleotide biosynthetic process, nucleotide biosynthetic process, pyrimidine nucleoside metabolic process, and pyrimidine-containing compound metabolic process (Figure 3E). Similarly, GO analysis of differentiated KC asterisk revealed enrichment in nucleotide-sugar metabolic process, nucleotide biosynthetic process, and pyrimidine nucleoside metabolic process, among others (Figure 3F). These findings support the involvement of both KC subsets in nucleotide and pyrimidine metabolism.

To further characterize the association between nucleotide or pyrimidine metabolism and disease severity, we performed correlation analysis between metabolite levels and clinical scores (PASI and BSA). Four metabolites including adenosine 5'-monophosphate, cytidine 3'-monophosphate, uridine, and 2'-deoxyadenosine-5'-monophosphate, showed significant positive correlations with both PASI and BSA scores (Figure S5A and B). The ROC curve analysis further demonstrated that these metabolites exhibited high AUC values, supporting their potential as diagnostic indicators for psoriasis (Figure S5C).

Additional metabolites showed partial correlations with clinical scores. For instance, 2'-deoxyinosine correlated significantly with PASI scores but not with BSA scores. Conversely, 2'-deoxyadenosine and xanthosine-5'-monophosphate correlated significantly with BSA scores but not with PASI scores (Figure S5D and E). These findings collectively highlight the complex relationship between nucleotide or pyrimidine metabolism and psoriasis severity, and identify specific metabolites and KC subsets as potential contributors to disease pathogenesis.

### Identification of metabolites associated with disease severity

**Figure 4:**
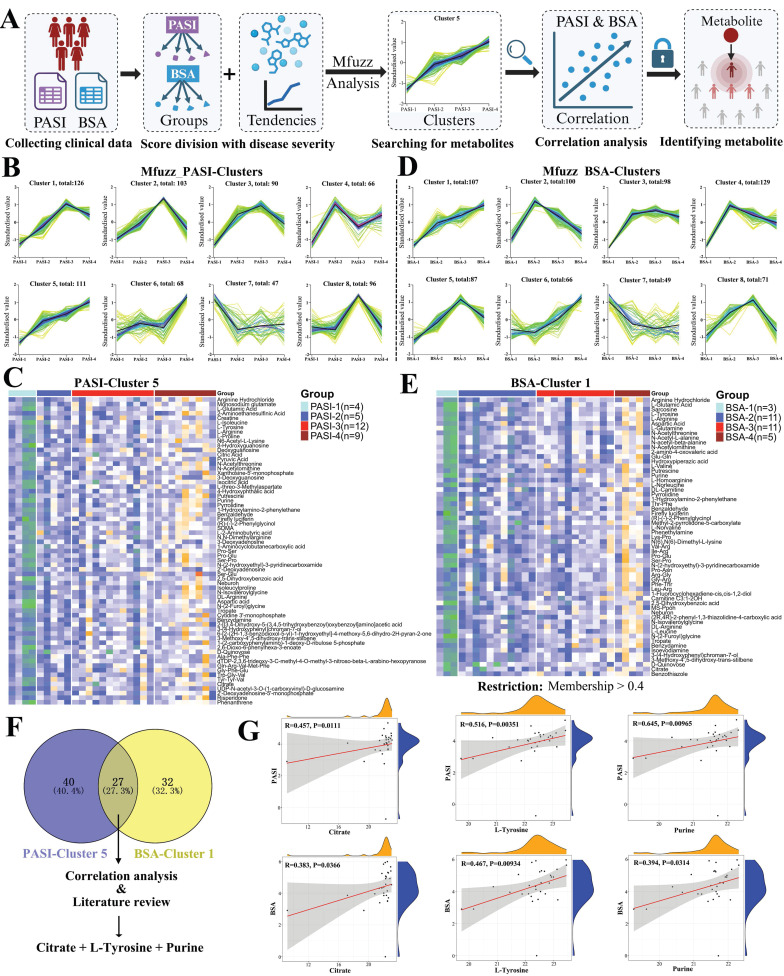
** Identification of metabolites associated with disease severity.** (A) Workflow for Mfuzz clustering analysis. (B) Mfuzz clustering analysis based on PASI scores identified eight clusters, with Cluster 5 showing a strong positive correlation with increasing PASI severity. (C) Heat map of 67 metabolites from PASI-Cluster 5 (membership > 0.4). (D) Mfuzz clustering analysis based on BSA scores identified eight clusters, with Cluster 1 positively correlated with BSA severity. (E) Heat map of 59 metabolites from BSA-Cluster 1 (membership > 0.4). (F) Venn diagram showing 27 overlapping metabolites between PASI-Cluster 5 and BSA-Cluster 1. (G) Correlation analyses of citrate, L-tyrosine, and purine with PASI and BSA scores.

Having identified hundreds of differential metabolites across multiple classes, we next sought to determine which groups or clusters of metabolites might synergistically reflect disease severity in psoriasis. To this end, we performed Mfuzz clustering analysis, which classifies metabolites based on their expression patterns across samples, enabling the identification of co-regulated metabolite groups and providing insights into their potential interrelationships and shared biological functions (Figure 4A).

For correlation with PASI scores, patients were stratified into four severity groups: PASI-1 (0-10), PASI-2 (10-15), PASI-3 (15-20), and PASI-4 (≥ 20). Mfuzz analysis generated eight distinct clusters based on metabolite expression patterns across these groups (Figure 4B). Among them, only Cluster 5, comprising 111 metabolites, exhibited a consistently positive correlation with increasing PASI severity. After applying a membership threshold greater than 0.4, 67 metabolites from this cluster were retained and visualized in a heat map (Figure 4C). A similar approach was applied to BSA scores, with patients divided into BSA-1 (0-10), BSA-2 (10-20), BSA-3 (20-40), and BSA-4 (≥ 40). Mfuzz analysis again yielded eight clusters, of which Cluster 1, containing 107 metabolites, showed a strong and consistently positive correlation with BSA severity (Figure 4D). Following the same membership filter (> 0.4), 59 metabolites were retained and are presented in Figure 4E.

To identify metabolites commonly associated with both clinical severity measures, we intersected the metabolite sets from PASI-Cluster 5 and BSA-Cluster 1. This combined analysis yielded 27 overlapping metabolites (Table S13). Based on correlation strength, consistency with both PASI and BSA scores, and literature review, we selected three metabolites including citrate, L-tyrosine, and purine for further validation as shown in the Venn diagram (Figure 4F). All three metabolites showed significant positive correlations with both PASI and BSA scores, supporting their potential as indicators for assessing psoriasis severity (Figure 4G).

### Validation of metabolite citrate, L-tyrosine, and purine with IMQ-induced psoriasis-like dermatitis mouse model

**Figure 5:**
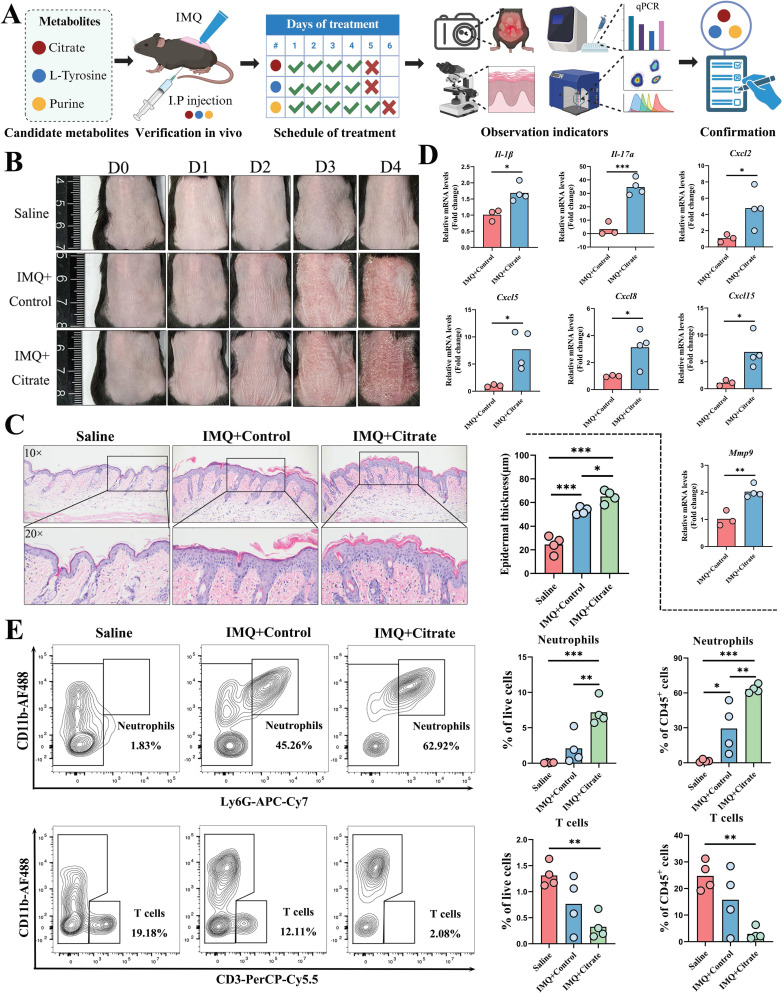
** Validation of citrate in an IMQ-induced psoriasis-like dermatitis mouse model.** (A) Experimental workflow for in vivo metabolite validation. (B) Citrate treatment exacerbated erythema and scaling in psoriatic lesions. (C) H&E staining showing increased epidermal thickness on day 4 in the citrate-treated group compared to IMQ+Control. (D) qPCR analysis revealed that citrate upregulated mRNA expression of *Il-1β*, *Il-17*, *Cxcl2*, *Cxcl5*, *Cxcl8*, *Cxcl15*, and *Mmp9*. (E) Flow cytometry showed increased neutrophil proportions in both live and CD45⁺ cells, with a compensatory decrease in T cells, following citrate treatment.

To functionally validate whether citrate, L-tyrosine, and purine could contribute to the pathogenesis of psoriasis, we employed an IMQ-induced psoriasis-like dermatitis mouse model and administered each metabolite via intraperitoneal injection. The experimental workflow was illustrated in Figure 5A.

Mice treated with citrate exhibited aggravated erythema and scaling of psoriatic lesions, accompanied by reduced body weight compared to controls (Figure 5B and Figure S6A). Histological analysis by H&E staining revealed a significant increase in epidermal thickness on day 4 (Figure 5C). And qPCR analysis of lesional skin showed that citrate upregulated the mRNA expression of pro-inflammatory cytokines *Il-1β*, *Il-17a*, *Cxcl2*, *Cxcl5*, *Cxcl8*, *Cxcl15*, and *Mmp9*, while expression levels of *Il-6*, *Il-22*, *Il-23*, *Icam1*, *Cxcl1*, *Ccl20*, *Tnf*, and *Ifn-γ* remained unchanged (Figure 5D and Figure S6B). Flow cytometric analysis of single-cell suspensions from skin lesions (gating strategy shown in Figure S6C) revealed that citrate treatment increased the proportion of CD45⁺ cells among live cells (Figure S6D) and neutrophils (Ly6G⁺CD11b⁺) among both live cells and CD45⁺ cells, while the proportion of T cells (CD3⁺CD11b⁻) was compensatorily reduced (Figure 5E). Proportions of LCs were also decreased in both live and CD45⁺ cell populations. Among CD45⁺ cells, macrophages and DCs were significantly reduced following citrate treatment (Figure S7A-C), whereas other CD11b⁺ cell populations remained unaffected (Figure S7D).

**Figure 6:**
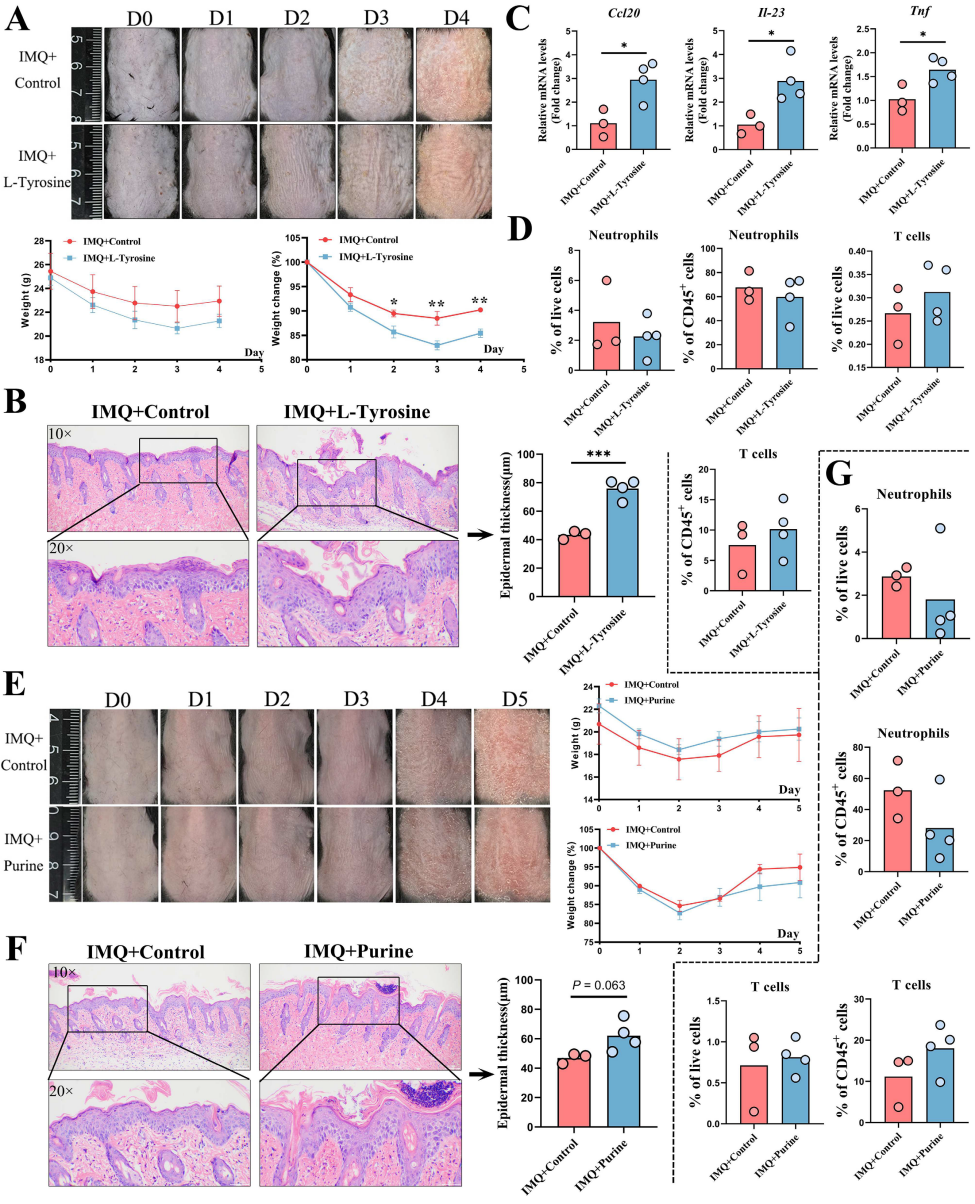
** Validation of L-tyrosine and purine in an IMQ-induced psoriasis-like dermatitis mouse model.** (A) L-tyrosine treatment exacerbated erythema and scaling and reduced body weight. (B) H&E staining showed increased epidermal thickness after L-tyrosine treatment. (C) qPCR analysis revealed significant upregulation of *Ccl20*, *Il-23*, and *Tnf* mRNA following L-tyrosine treatment. (D) Flow cytometry showed no significant changes in neutrophil or T cell proportions after L-tyrosine treatment. (E) Purine treatment did not significantly affect erythema, scaling, or body weight. (F) H&E staining showed no significant increase in epidermal thickness with purine treatment (P = 0.063). (G) Flow cytometry revealed no significant changes in neutrophil or T cell proportions after purine treatment.

Mice treated with L-tyrosine for four consecutive days similarly exhibited aggravated erythema and scaling, along with reduced body weight (Figure 6A). Epidermal thickness was significantly increased, as assessed by H&E staining (Figure 6B). And qPCR analysis revealed significant upregulation of *Ccl20*, *Il-23*, and *Tnf* mRNA expression (Figure 6C), while *Cxcl1*, *Cxcl2*, *Cxcl8*, *Ifn-γ*, *Il-1β*, and *Il-17a* showed no significant changes (Figure S8A). In contrast to the findings with citrate, flow cytometry revealed no significant alterations in the proportions of neutrophils, T cells (Figure 6D), LCs, macrophages, DCs, or other CD11b⁺ cells in either live or CD45⁺ cell populations following L-tyrosine treatment (Figure S8B).

Unlike citrate and L-tyrosine, purine treatment did not significantly affect the severity of erythema, scaling, or body weight in IMQ-treated mice (Figure 6E). Epidermal thickness showed a trend toward increase but did not reach statistical significance (P = 0.063, Figure 6F). No significant changes were observed in the mRNA expression levels of any cytokines assessed (Figure S8C). Flow cytometric analysis revealed no significant differences in the proportions of neutrophils, T cells (Figure 6G), DCs, LCs, or macrophages (Figure S8D) following purine treatment.

In summary, citrate and L-tyrosine significantly exacerbated IMQ-induced psoriasis-like dermatitis, whereas purine showed no obvious pathogenic effects. These findings support the potential roles of citrate and L-tyrosine as contributors to psoriasis severity and as candidate therapeutic targets.

## Discussion

Psoriasis is a complex multifactorial disease involving genetic predisposition, immune dysregulation, and environmental triggers. Metabolomics enables a comprehensive assessment of metabolic alterations in patients, offering insights into pathogenic pathways and providing new clues for understanding disease mechanisms. Abnormal metabolic patterns may be linked to key pathological processes such as immune cell activation, inflammatory mediator release, and epidermal hyperproliferation. Evidence have indicated that psoriasis can also drive the expansion of IL-1β-producing intestinal macrophages that may impair chylomicron secretion and promote epithelial lipid accumulation, establishing a macrophage-mediated metabolic link between psoriasis and gut inflammation 37. Previous studies have demonstrated that high-fat diet (HFD)-induced obesity exacerbates psoriasis by amplifying IL-17 signaling through branched-chain amino acid (BCAA) catabolism mediated by branched-chain aminotransferase 2 (BCAT2), with elevated levels of leucine, isoleucine, and valine 38. Conversely, quinolinic acid, a skin microbiota-derived metabolite, was shown to alleviate skin inflammation in an AhR-dependent manner by inhibiting NLRP3 inflammasome activation 39. In the context of immune modulation, inhibition of sphingosine-1-phosphate (S1P) signaling via ceramidase and sphingosine kinase 2 inhibitors blocked Th17 cell differentiation from naïve CD4^+^ T cells, thereby alleviating IMQ-induced skin lesions and reducing serum IL-17A levels 40. Moreover, D-mannose was reported to downregulate HIF-1α and CCL20 expression in KCs, suppress Th17 infiltration, and disrupt the KC-Th17 feedback loop 41. Furthermore, Mfsd2a can mediate the uptake of plasma-derived lysophosphatidylcholine (LPC) by epidermal KCs, and its deficiency can disrupt phospholipid homeostasis, impair KC differentiation, and lead to dermatitis, which reveals a critical pathway for lipid acquisition required for skin barrier maintenance and repair 42. Abnormal lipid metabolism can linked with ferroptosis in psoriasis since fatty-acid binding protein 5 (FABP5) can promote psoriatic skin inflammation by suppressing the ferroptosis regulator Gpx4, and inhibiting FABP5 or ferroptosis can actually ameliorate the skin phenotype in a preclinical mouse model 43. Collectively, these findings underscore the significance of metabolomics in elucidating psoriasis pathogenesis, supporting disease diagnosis and differential diagnosis, enabling disease assessment and prognosis, guiding personalized treatment strategies, and facilitating the development of novel therapeutics.

In the present study, we performed TM wide-targeted metabolomic profiling of skin lesions from psoriasis patients and identified 707 differentially abundant metabolites spanning 21 Class I categories. Unlike prior serum-based or small-scale skin metabolomics studies, our approach provides a comprehensive view of the lesional metabolic landscape, which is particularly relevant given the propensity for in situ recurrence in psoriasis 29, 44. KCs in psoriatic lesions exhibit accelerated proliferation and differentiation, and even during remission, these cells may retain inflammatory memory 45, 46. Upon exposure to triggers such as skin injury, infection, or chemical stimuli, KCs can reinitiate abnormal proliferation and differentiation, leading to local relapse. By directly targeting lesional tissue, our metabolomic analysis more accurately reflects site-specific metabolic alterations and their relationship to local disease mechanisms. Among the identified differential metabolites, cetylpyridinium chloride (CPC) was previously shown to induce cell death and enhance pulmonary inflammatory cytokine secretion via mitochondrial damage and iron depletion 47. Kynurenine, a tryptophan metabolite upregulated in psoriatic lesions and regulated by L-kynureninase (KYNU), was found to increase cytokine and chemokine expression and promote cell adhesion 48, 49. Elevated serum uric acid levels in psoriasis patients, which significantly decreased after 48 weeks of secukinumab treatment, further support the involvement of purine metabolism 50. Enhanced glycolysis, tricarboxylic acid (TCA) cycle activity, and lactate transport have also been reported, particularly in differentiated KCs within psoriatic lesions 51. Through targeting TCA cycle, a hydrogen-releasing microbubble hydrogel enables topical psoriasis therapy by suppressing KC hyperproliferation via PKM2-mediated anti-Warburg metabolic reprogramming and restoring redox homeostasis 52. Additionally, we distinguished between endogenous and exogenous metabolites to provide a more comprehensive understanding of cellular metabolic processes and environmental influences, thereby offering valuable insights for diagnosis, treatment, and prevention.

Nucleotide and pyrimidine metabolism emerged as overlapping pathways in KEGG enrichment analyses of both our metabolomic data and differentially expressed genes in psoriatic lesions. Nucleotides are fundamental building blocks of DNA and RNA, and their metabolism, including synthesis, degradation, and interconversion, is tightly linked to cellular proliferation. In psoriasis, epidermal hyperproliferation demands increased nucleotide synthesis to support DNA replication. Dysregulated nucleotide metabolism may therefore perturb normal cell cycle control and promote aberrant KC expansion. Moreover, intermediates and enzymes within these pathways can modulate immune cell activation, differentiation, and effector functions, contributing to the immune imbalance characteristic of psoriasis. Given the elevated metabolic activity and energy demands of lesional cells, alterations in nucleotide metabolism may also affect energy homeostasis and cellular function. For instance, silencing or inhibition of serine hydroxymethyltransferase (SHMT), which converts serine to glycine and tetrahydrofolate-bound one-carbon units, suppressed KC proliferation and inflammatory cell expansion via purine depletion 53. Cholera toxin (CT), a potent cAMP inducer, was shown to produce a more pronounced psoriatic phenotype than other agents, resulting in epidermal thickening and altered expression of involucrin, filaggrin, and keratin 10, suggesting that cAMP upregulation may contribute to disease severity 54.

Pyrimidine metabolism is particularly critical for nucleic acid synthesis and cell division. In psoriasis, excessive KC proliferation requires abundant pyrimidines to sustain DNA replication. Pyrimidine metabolic disturbances may also influence immune cell function, exacerbating inflammatory responses. Genes such as *CMPK2* and *RRM2*, identified as differentially expressed in our bioinformatic analysis, have been implicated in pyrimidine metabolism 55. Notably, several pyrimidine-based therapeutic strategies have been explored, including CXCR2-specific small molecule inhibitors and computational drug repurposing for psoriasis 56, 57. These findings highlight the potential of targeting nucleotide and pyrimidine metabolism for novel therapeutic interventions.

Based on their strong correlations with PASI and BSA scores, representation of distinct key metabolic pathways, including the TCA cycle (citrate), amino acid metabolism (L-tyrosine), and nucleotide metabolism (purine), literature support for their potential as actionable targets, and the need to include both positive and negative controls for subsequent studies, citrate, L-tyrosine, and purine were selected from the 27 overlapping metabolites for in vivo validation. Among the metabolites validated in our study, citrate, a key TCA cycle intermediate, was found to accumulate in mitochondria during LPS-induced acute lung injury via excessive mitophagy-mediated necroptosis 58. Dietary citrate supplementation in mice can induce hyperinsulinemia and insulin resistance, accompanied by elevated hepatic inflammatory markers 59. Our group previously reported that insulin resistance is positively associated with fatty liver disease in psoriasis and may impair the efficacy of biologic therapy in moderate-to-severe plaque psoriasis 60, 61. What is more, microglial IDH1 can drive Alzheimer's pathology by disrupting citrate metabolism and mitochondrial TCA function, and its inhibition by Kinsenoside can restore metabolic homeostasis, reduces Aβ deposition and neuroinflammation, and improve cognition 62. L-tyrosine, an aromatic amino acid, serves as a substrate for tyrosine kinase, and tyrosine phosphorylation is a critical signaling mechanism regulating cell proliferation, differentiation, and migration. Deucravacitinib, a selective allosteric TYK2 inhibitor, has demonstrated superior efficacy to placebo or apremilast in treating moderate-to-severe scalp psoriasis with a favorable safety profile 63, 64. Recent study has revealed that food additives such as benzoic acid, dehydroacetic acid, and acesulfame may disrupt CD4^+^ T cell metabolic homeostasis, particularly affecting tyrosine metabolism along with phenylalanine and tryptophan biosynthesis to thereby aggravate airway inflammation and immune imbalance in childhood asthma 65. Purine, involved in energy transfer, signaling, and coenzyme formation, has been linked to metabolic syndrome components such as obesity, diabetes, and dyslipidemia, which are common comorbidities in psoriasis 7, 66. Integrated proteomics and metabolomics analyses have revealed that phosphatidylethanolamine (PE) supplementation in macrophages can attenuate LPS-induced inflammation by reinforcing glutathione metabolism and inducing context-dependent shifts in purine and amino-acid pathways, thereby supporting a restrained inflammatory phenotype 67. These observations support the rationale for dietary and lifestyle interventions targeting metabolic disturbances in psoriasis management. Although we found that differential metabolites in psoriatic skin lesions were significantly enriched in nucleotide metabolism pathways, our in vivo validation experiments did not observe any effect of purine supplementation on the psoriasis-like dermatitis phenotype. On the one hand, we consider that insufficient dosage, low dosing frequency, and the vehicle may have affected bioavailability. On the other hand, the balance of adenosine and purinergic receptor signaling is bidirectional, and the acute TLR7-driven inflammatory signal induced by IMQ may be far stronger than any modulatory effect of purine 68. Moreover, since our initial metabolite screening was based on human samples, it is possible that significant species differences in purine metabolism and enzyme activity between mice and humans prevent purine from reaching effective tissue concentrations. Alternatively, the altered purine metabolites may simply be an epiphenomenon associated with disease severity or even a protective factor rather than a direct pathogenic driver, as recently reported for rosacea, where serum levels of α-ketoglutarate (α-KG) were elevated and correlated positively with erythema severity, but further results indicated that the increase in α-KG might represent a compensatory protective response 69. Therefore, the impact of purine metabolites on psoriasis warrants further investigation.

This study has several limitations. First, the sample size was relatively modest, and due to the inherent difficulty in obtaining skin lesion biopsies, we were unable to perfectly match the healthy control group for demographic variables such as gender, age, and body mass index (BMI). Second, our metabolomic approach provided only relative quantification of differential metabolites without absolute quantification using authentic standards. Third, many of the identified metabolites remain poorly characterized, and some are relatively uncommon, posing challenges for subsequent validation experiments. Additionally, we did not perform targeted mass spectrometric detection and quantitative analysis for each metabolite of interest, and several metabolic pathways containing differential metabolites warrant further investigation. Future studies with larger cohorts, absolute quantification, and functional validation are needed to confirm and extend our findings.

## Conclusions

In summary, this study provides a comprehensive metabolomic profiling of skin lesions from patients with psoriasis versus healthy controls, identifying 707 differentially abundant metabolites spanning nearly all major classes, including amino acids and their metabolites, fatty acids, organic acids and derivatives, nucleotides and their metabolites, and benzene and substituted derivatives. Integrative analysis of bulk and scRNA-seq data revealed that differentiated KCs may regulate nucleotide and pyrimidine metabolism through key differentially expressed genes such as *UPP1*, *TYMP*, *RRM2*, *PNP*, *GDA*, *CMPK2*, *TK1*, *NT5C3A*, and *AMPD3*. Mfuzz clustering analysis identified 27 metabolites common to PASI-Cluster 5 and BSA-Cluster 1, including citrate, L-tyrosine, and purine, which positively correlated with disease severity. Validation in an IMQ-induced psoriasis-like dermatitis mouse model confirmed that citrate and L-tyrosine may serve as potential diagnostic indicators and therapeutic targets for psoriasis. Collectively, our findings uncover metabolic signatures associated with disease severity and offer new insights for therapeutic strategies in psoriasis.

## Supplementary Material

Supplementary figures and tables.

## Figures and Tables

**Table 1:** Clinical characteristics of the study participants

Characteristics	Psoriasis patients (PS)	Healthy control subjects (HC)	*P* value
Number	30	30	1
Sex (male/female)	23/7	17/13	0.10
Age in years	43.53 ± 12.62	43.87 ± 19.93	0.94
PASI score	17.36 ± 7.66	-	-
BSA score	25.84 ± 16.48	-	-

## Data Availability

The raw data of metabolomics underlying this article cannot be shared publicly due to protect the privacy of individuals that participated in the study. Other data that support the findings of this study are available from the corresponding author upon reasonable request.

## Abbreviations

AUC: area under curve
BSA: body surface area
DC: dendritic cell
IMQ: imiquimod
KC: keratinocyte
LC: langerhans cell
OPLS-DA: orthogonal partial least squares-discriminant analysis
PASI: psoriasis area and severity index
ROC: receiver operating characteristic
TCA cycle: tricarboxylic acid cycle
VIP: variable importance in projection
