# Supplementary material for: Comprehensive Metabolomic Profiling of Skin Lesions from Psoriasis Patients Reveals Disease Signatures
Source: Int J Biol Sci. 2026 May 29;22(11):5934–52. doi: 10.7150/ijbs.134115 (PMC13282745; doi:10.7150/ijbs.134115)
Supplement: Supplementary file 1 — Supplementary figures and tables. [file ijbsv22p5934s1.pdf]

Supplementary files

Comprehensive Metabolomic Profiling of Skin Lesions from Psoriasis Patients Reveals Disease Signatures

Supplementary figures

- Figure S1. Overview of TM widely-targeted metabolomics.
- Figure S2. Violin plots, correlation analyses, and ROC curves of the remaining top 10 endogenous differential metabolites.
- Figure S3. Violin plots, correlation analyses, and ROC curves of the top 10 exogenous differential metabolites.
- Figure S4. Comprehensive KEGG analysis of metabolomics and differential genes from the GSE13355 database.
- Figure S5. Correlation between nucleotide or pyrimidine metabolites and clinical severity scores (PASI and BSA).
- Figure S6. Cytokine mRNA expression following citrate treatment and flow cytometry gating strategy.
- Figure S7. Proportions of LCs, macrophages, DCs, and other $CD11b^+$ cells in mouse skin lesions following citrate treatment.
- Figure S8. Cytokine expression and immune cell infiltration following L-tyrosine and purine treatment.

Supplementary tables

- Table S1. Top 20 differential metabolites among amino acid and its metabolites (Class I).
- Table S2. Top 20 differential metabolites among FA (Class I).
- Table S3. Top 20 differential metabolites among organic acid and its metabolites (Class I).
- Table S4. Top 20 differential metabolites among nucleotide and its metabolites (Class I).
- Table S5. Top 20 differential metabolites among benzene and substituted derivatives (Class I).
- Table S6. Top 20 differential metabolites among heterocyclic compounds (Class I).
- Table S7. Top 20 differential metabolites among carbohydrates and its metabolites (Class I).
- Table S8. Top 20 differential metabolites among GP (Class I).
- Table S9. Top 20 differential metabolites among alcohol and amines (Class I).
- Table S10. Differential metabolites among aldehyde, ketones, esters (Class I).
- Table S11. Top 20 differential metabolites among Others.
- Table S12. Top 20 differential metabolites among small peptide (Class II).
- Table S13. Overlaps of differential metabolites between PASI-Cluster 5 and BSA-Cluster 1.

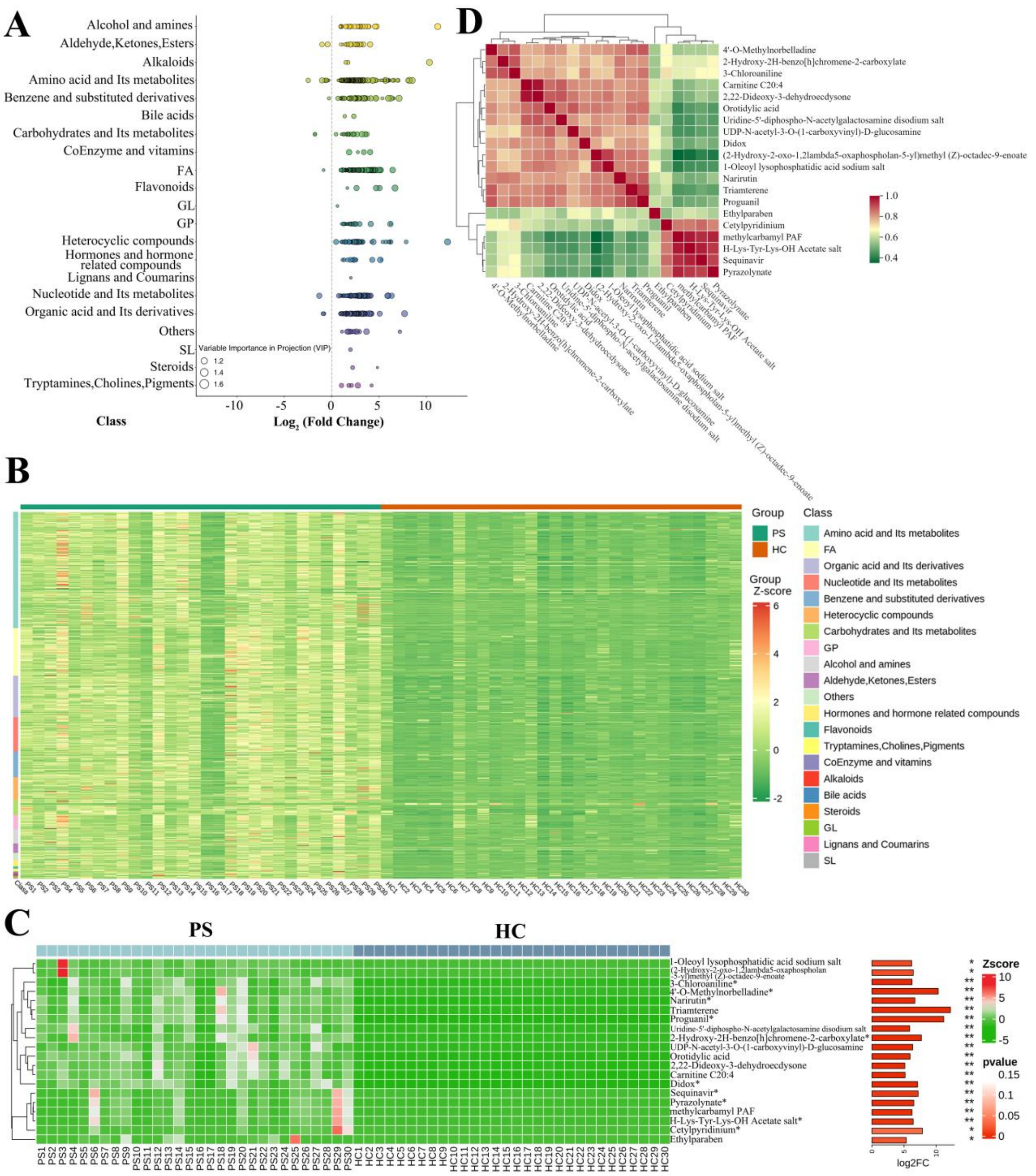

Figure S1. Overview of TM widely-targeted metabolomics. (A) Scatter plot showing differences in relative abundance of various metabolite classes between psoriasis patient (PS) and healthy control (HC) samples. (B) Heat map of all differentially abundant metabolites between PS and HC. (C) Heat map of the top 10 endogenous and exogenous differential metabolites after filtering out small peptides. (D) Correlation heat map of the top 10 endogenous and exogenous differential metabolites (small peptides excluded).

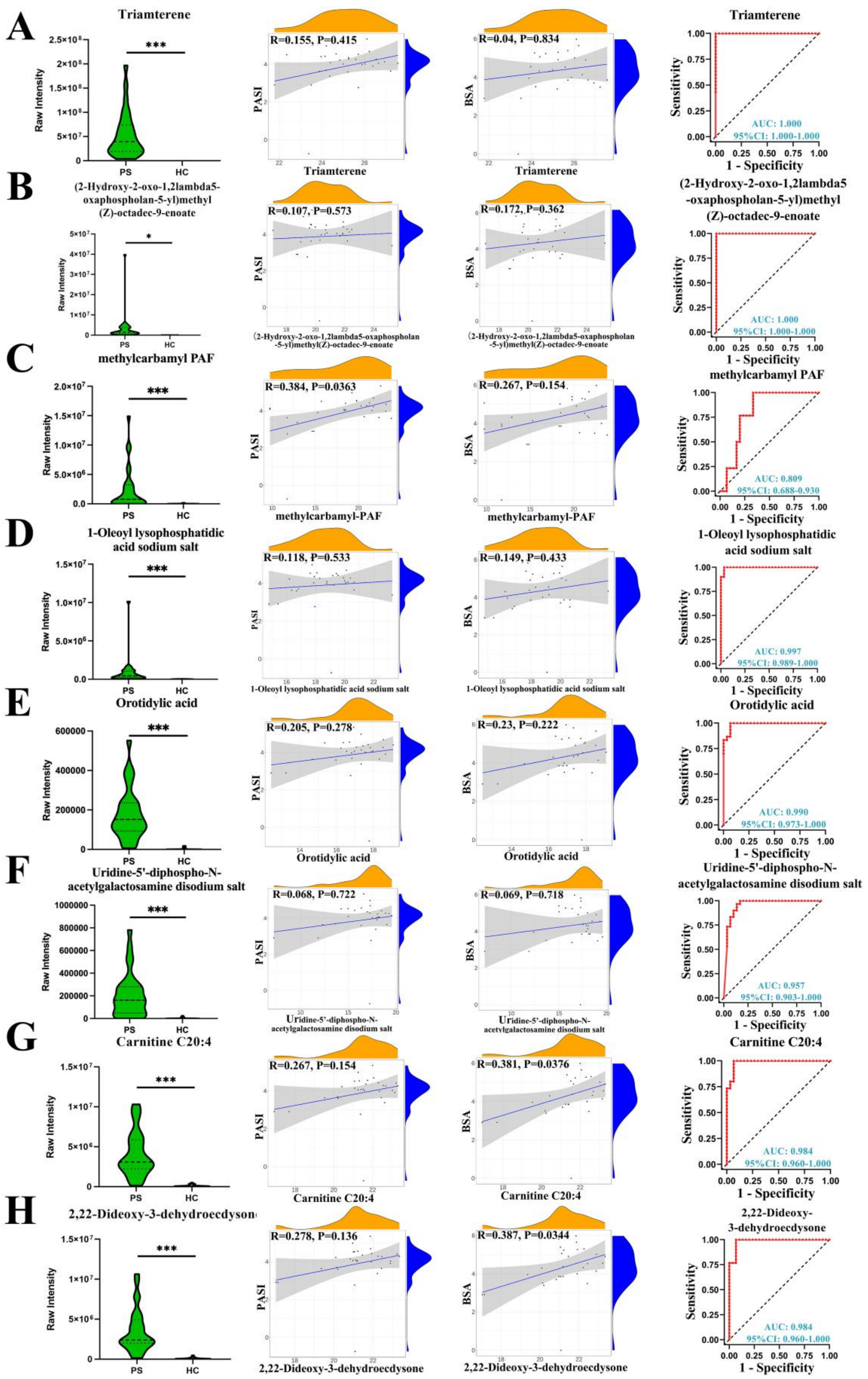

Figure S2. Violin plots, correlation analyses, and ROC curves of the remaining top 10 endogenous differential metabolites. (A-H) Violin plots, correlation with PASI and BSA scores, and ROC curves for: Triamterene, (2-hydroxy-2-oxo-1,2lambda5-oxaphospholan-5-yl) methyl (Z)-octadec-9-enoate, methylcarbamyl PAF, 1-oleoyl lysophosphatidic acid sodium salt, orotidylic acid, uridine-5'-diphospho-N-acetylgalactosamine disodium salt, carnitine C20:4, and 2,22-dideoxy-3-dehydroecdysone.

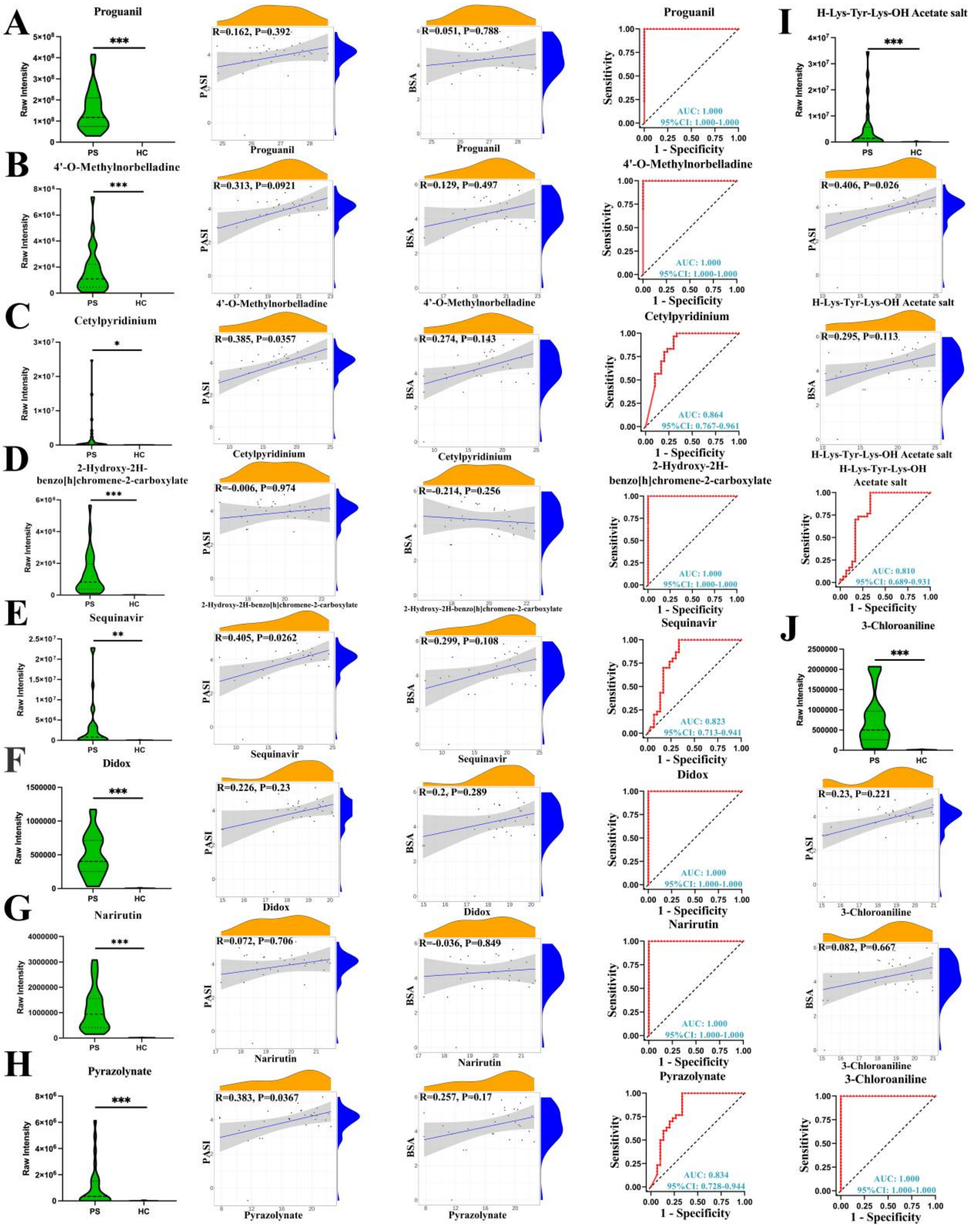

Figure S3. Violin plots, correlation analyses, and ROC curves of the top 10 exogenous differential metabolites. (A-J) Violin plots, correlation with PASI and BSA scores, and ROC curves for: Proguanil, 4'-O-Methylnorbelladine, Cetylpyridinium, 2-Hydroxy-2H-benzo[h]chromene-2-carboxylate, Sequinavir, Didox, Narirutin, Pyrazolynate, H-Lys-Tyr-Lys-OH Acetate salt, and 3-Chloroaniline.

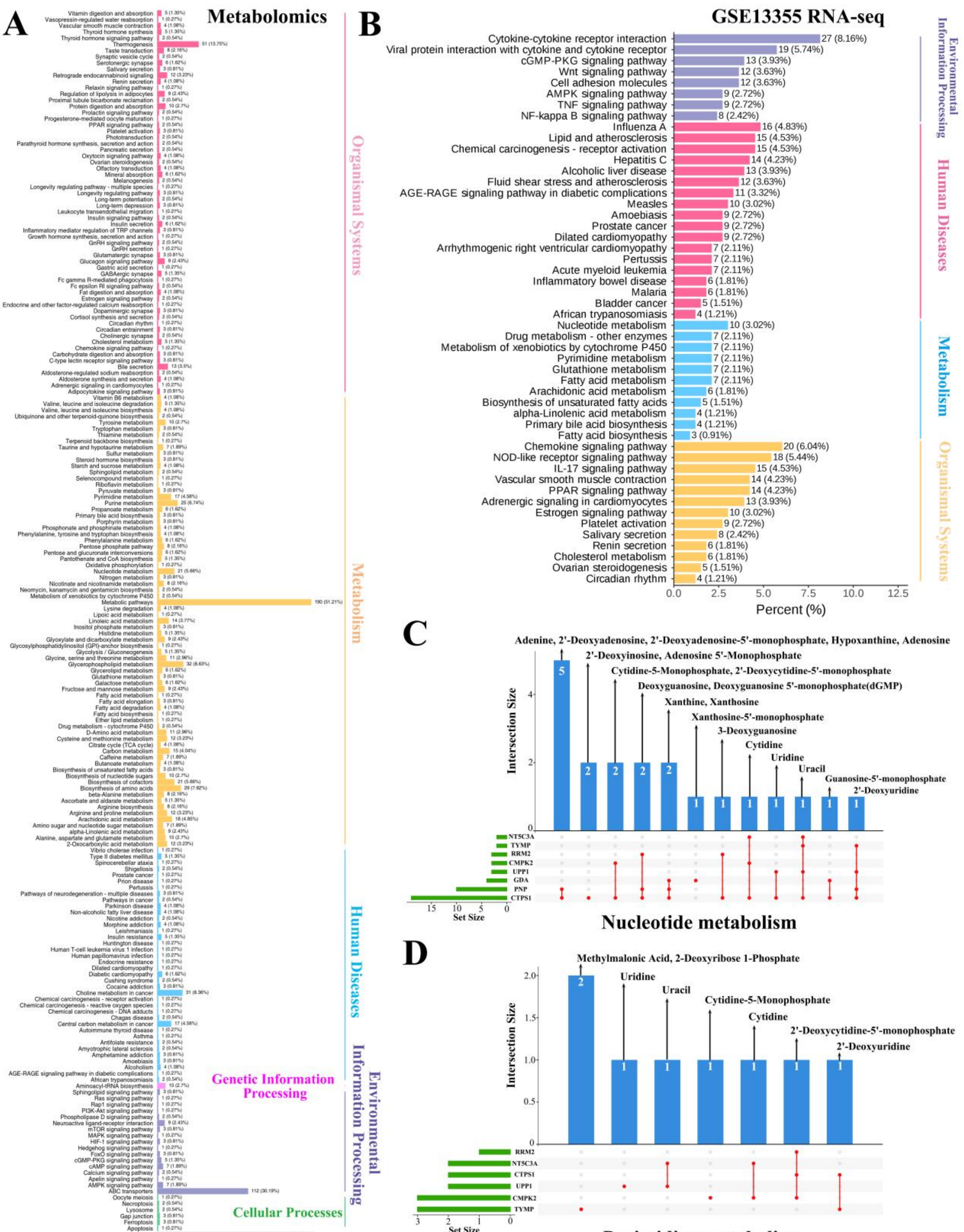

Figure S4. Comprehensive KEGG analysis of metabolomics and differential genes from the GSE13355 database. (A) KEGG pathway enrichment of all differential metabolites. (B) KEGG enrichment analysis of differentially expressed genes in the GSE13355 dataset. (C) UpSetR plot depicting regulatory genes involved in the production and conversion of corresponding metabolites, including *TK1*, *AMPD3*, *CTPS1*, *UPP1*, *TYMP*, *GDA*, *PNP*, *CMPK2*, *NT5C3A*, and *RRM2*. (D) UpSetR plot showing regulatory roles of *RRM2*, *NT5C3A*, *CTPS1*, *UPP1*, *CMPK2*, and *TYMP* in pyrimidine metabolism.

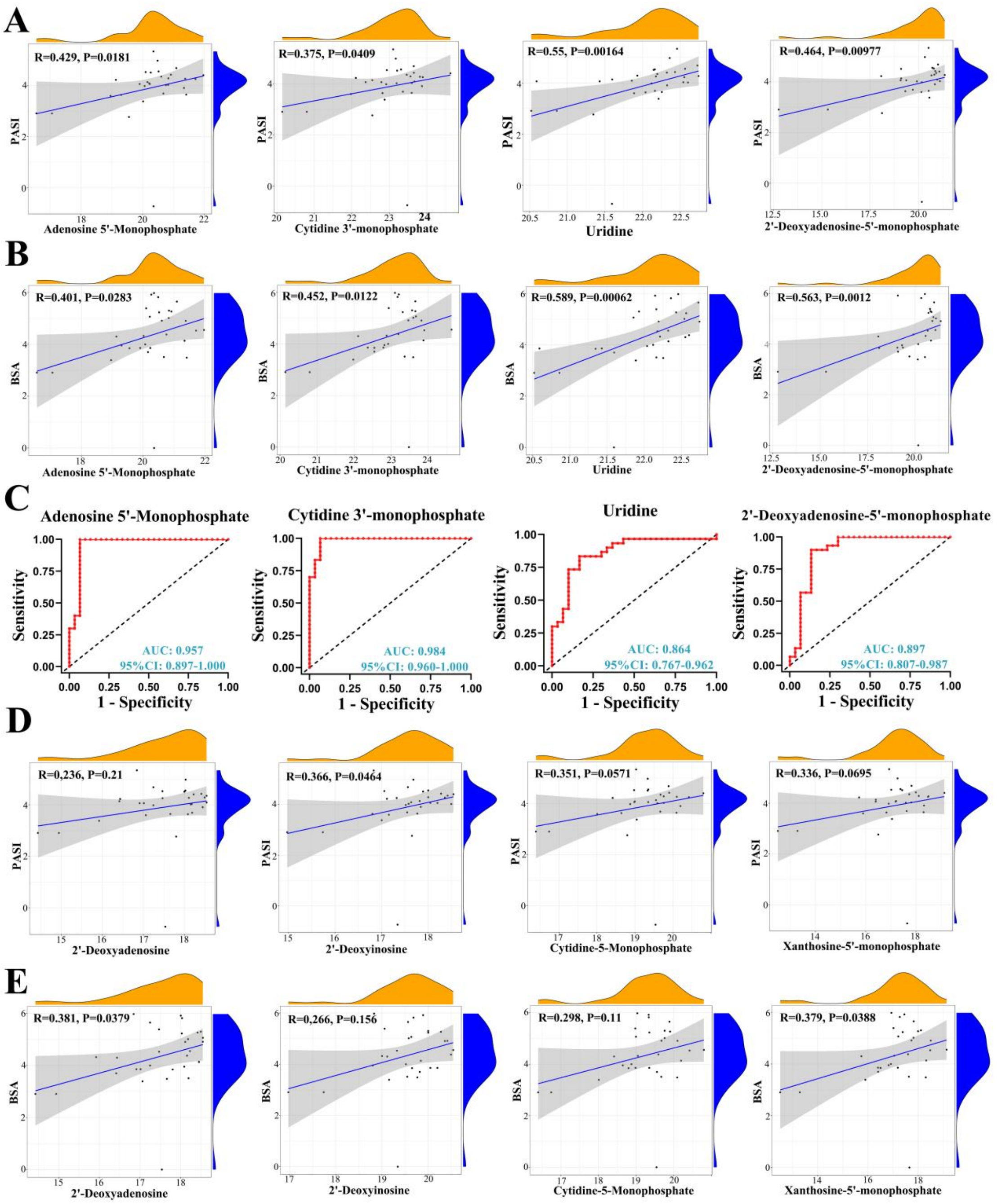

Figure S5. Correlation between nucleotide or pyrimidine metabolites and clinical severity scores (PASI and BSA). (A-B) Four differential metabolites within nucleotide and pyrimidine metabolism pathways showed significant positive correlations with both PASI and BSA scores. (C) ROC curves indicated that Adenosine 5'-Monophosphate, Cytidine 3'-monophosphate, Uridine, and 2'-Deoxyadenosine-5'-monophosphate may serve as potential indicators for psoriasis. (D-E) Correlation analysis of additional metabolites including 2'-Deoxyadenosine, 2'-Deoxyinosine, Cytidine-5-Monophosphate, and Xanthosine-5'-monophosphate with PASI and BSA scores.

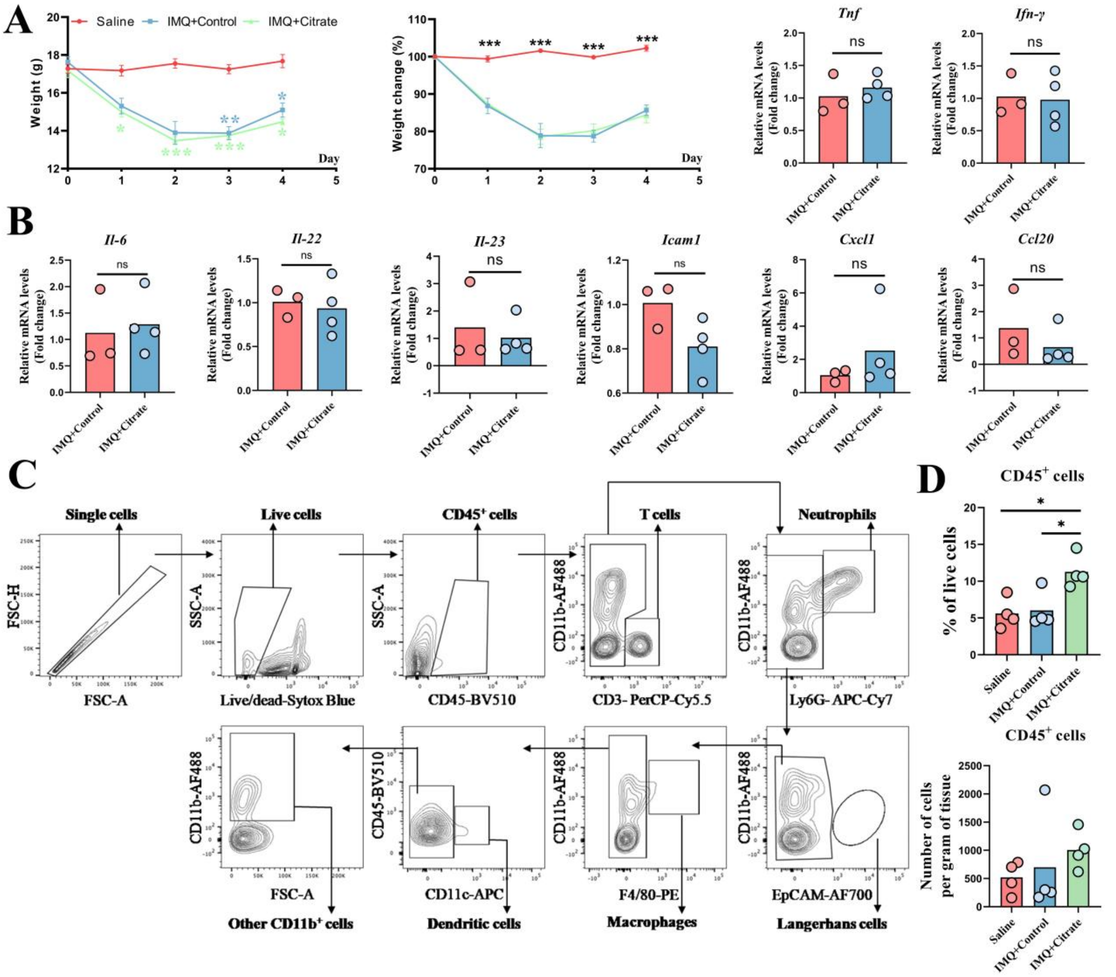

Figure S6. Cytokine mRNA expression following citrate treatment and flow cytometry gating strategy. (A) The change of body weight after treatment in mice. (B) Relative mRNA expression levels of cytokines showed no significant changes after citrate treatment. (C) Gating strategy for flow cytometric analysis of immune cell populations. (D) Citrate treatment increased the proportion of $CD45^{+}$ cells among live cells in skin lesions.

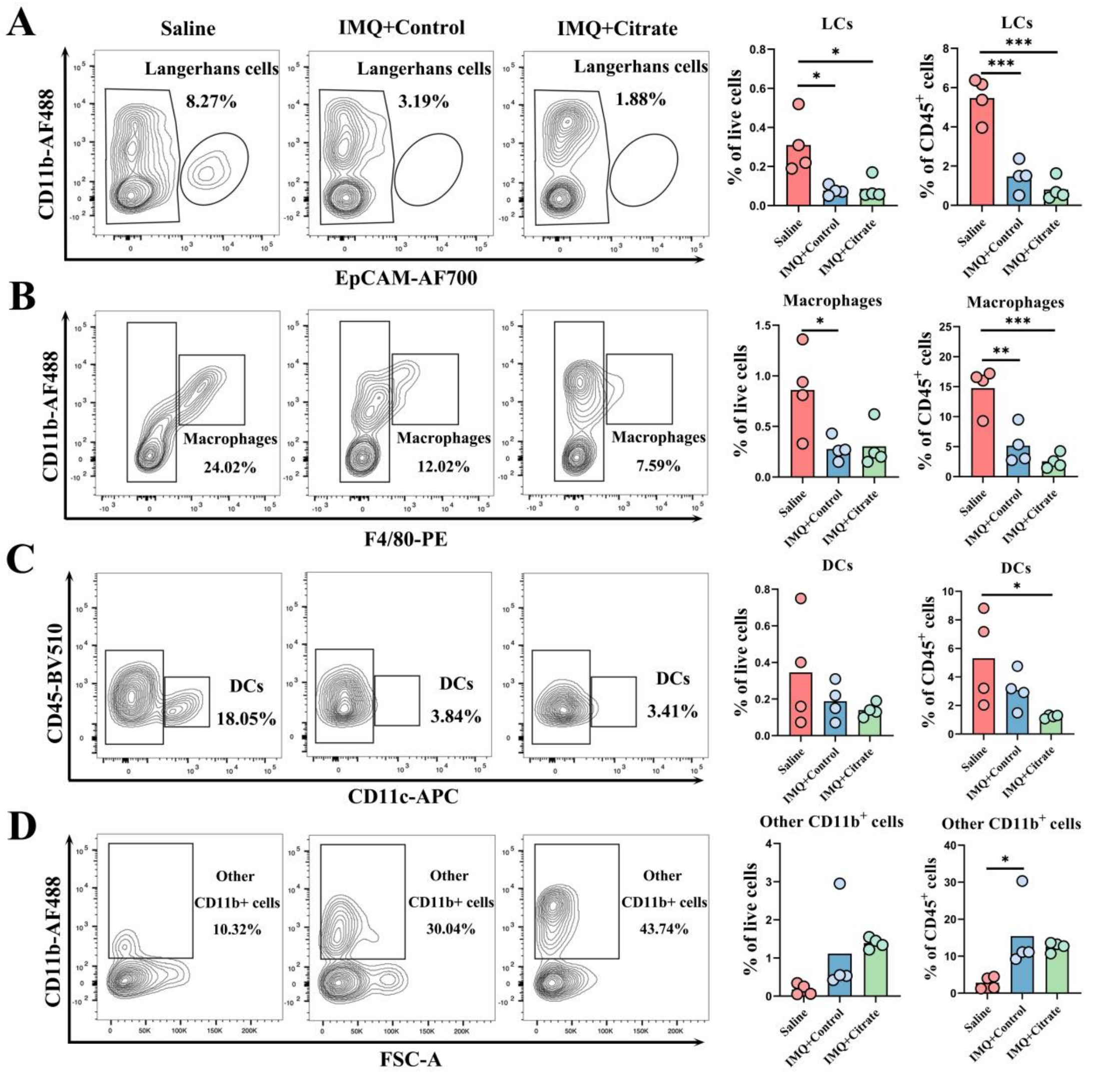

Figure S7. Proportions of LCs, macrophages, DCs, and other CD11b$^+$ cells in mouse skin lesions following citrate treatment. (A-D) Citrate treatment significantly decreased the proportions of Langerhans cells (LCs) in both live and CD45$^+$ cell populations, while macrophages and dendritic cells (DCs) were reduced specifically within the CD45$^+$ compartment. No significant changes were observed in other CD11b$^+$ cell subsets.

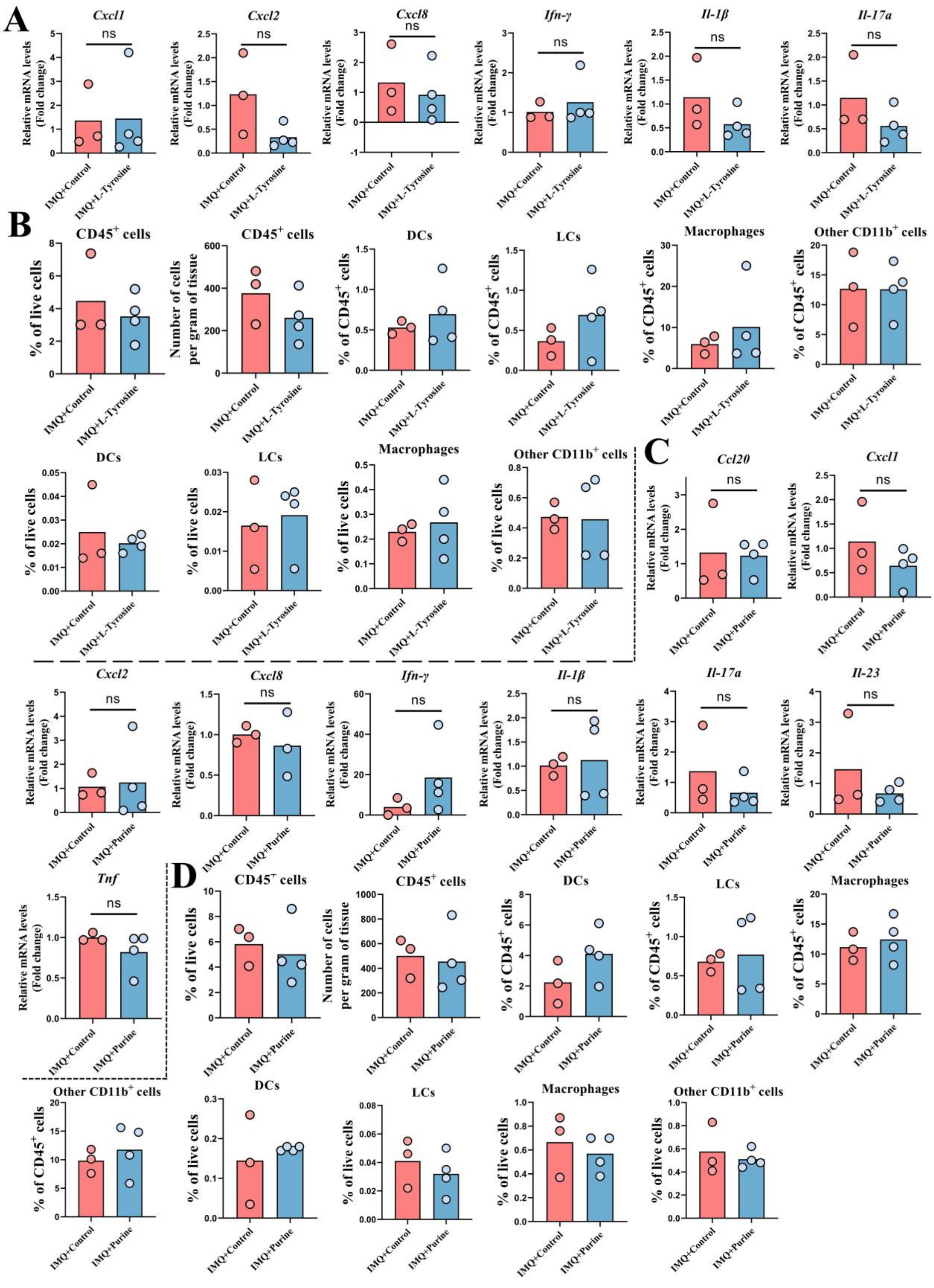

Figure S8. Cytokine expression and immune cell infiltration following L-tyrosine and purine treatment. (A) Relative mRNA expression levels of cytokines showed no significant changes after L-tyrosine treatment. (B) Proportions of LCs, macrophages, DCs, and other $CD11b^+$ cells in skin lesions following L-tyrosine supplementation. (C) No significant changes were observed in the mRNA expression levels of any assessed cytokines after purine treatment. (D) Proportions of DCs, LCs, and macrophages in skin lesions showed no significant changes following purine supplementation.

Table S1. Top 20 differential metabolites among amino acid and its metabolites (Class I)

Compounds	Class II	VIP	P-value	Log2FC	Regulation	Type*
7-Hydroxycoumarinyl Arachidonate	polypeptide	1.0661	0.0005	5.6717	up	B
Cyclopentylglycine	Amino acid derivatives	1.6544	0.0000	4.3182	up	-
1-Methylpiperidine-2-carboxylic acid	Amino acid derivatives	1.6544	0.0000	4.3182	up	-
N'-Formylkynurenine	Amino acid derivatives	1.4275	0.0008	3.8596	up	A
1,3-Dimethyluric acid	Amino acid derivatives	1.5677	0.0000	3.3399	up	A
Methionine Sulfoxide	Amino acid derivatives	1.5238	0.0000	3.0235	up	A
Phenylacetyl-L-Glutamine	Amino acid derivatives	1.4242	0.0000	2.9019	up	A
Aspartic acid	Amino acids	1.5164	0.0000	2.8215	up	A
D-Kynurenine	Amino acid derivatives	1.4634	0.0000	2.8179	up	A
O-Acetyl-L-homoserine	Amino acid derivatives	1.4592	0.0000	2.7444	up	A
O-Phospho-L-Serine	Amino acid derivatives	1.4228	0.0000	2.7266	up	A
Carnosine	Amino acid derivatives	1.1742	0.0000	2.6597	up	A
2-[(2-Amino-3-phenylpropanoyl)amino]-3-methylbutanoic acid	Amino acid derivatives	1.2579	0.0000	2.6291	up	B
N-Acetyl-L-methionine	Amino acid derivatives	1.5846	0.0000	2.5332	up	A
L-kynurenine	Amino acid derivatives	1.3744	0.0000	2.4467	up	A
L-Homoarginine	Amino acids	1.5921	0.0000	2.4022	up	A
DL-2-Methylglutamic acid	Amino acids	1.3860	0.0000	2.3373	up	A
N-Methylisoleucine	Amino acids	1.3433	0.0000	2.2366	up	-
Mimosine	Amino acids	1.4988	0.0000	2.1816	up	B
Pro-Phe	Amino acid derivatives	1.1250	0.0001	2.1534	up	A

*A: Endogenous metabolites; B: Exogenous metabolites; -: Unable to determine.

Table S2. Top 20 differential metabolites among FA (Class I)

Compounds	Class II #	VIP	P-value	Log2FC	Regulation	Type*
(2-Hydroxy-2-oxo-1,2lambda5-oxaphosp holan-5-yl)methyl (Z)-octadec-9-enoate	FFA	1.6331	0.0153	6.4393	up	A
Carnitine C20:4	CAR	1.5739	0.0000	5.1736	up	A
Carnitine ph-C14	CAR	1.6315	0.0000	5.1311	up	A
Carnitine C18:2	CAR	1.6394	0.0000	4.9325	up	A
Carnitine C18:3	CAR	1.6119	0.0000	4.6089	up	A
Carnitine C20:3	CAR	1.5829	0.0000	4.5521	up	A
Carnitine isoC4:0	CAR	1.6298	0.0000	4.4043	up	A
Carnitine C4:0	CAR	1.6298	0.0000	4.4043	up	A
Clupanodonyl carnitine	CAR	1.5763	0.0000	4.3703	up	A
Carnitine C16:1	CAR	1.5751	0.0000	4.2042	up	A
Carnitine C14:2:DC	CAR	1.5766	0.0000	4.1490	up	A
Carnitine C22:6	CAR	1.4715	0.0000	4.0930	up	A
Carnitine C22:5	CAR	1.5575	0.0000	4.0503	up	A
n-Oleoylethanolamine	Others	1.4530	0.0000	4.0355	up	B
2-Hydroxyhexadecanoic acid	FFA	1.4802	0.0001	3.9925	up	A
Carnitine C17:1	CAR	1.4984	0.0000	3.8893	up	A
Carnitine C18:1	CAR	1.6243	0.0000	3.8050	up	A
12-Methyltridecanal	FFA	1.4894	0.0000	3.7466	up	A
Carnitine-2-methyl-C4	CAR	1.5078	0.0000	3.4279	up	A
Carnitine C5:0	CAR	1.5078	0.0000	3.4279	up	A

*A: Endogenous metabolites; B: Exogenous metabolites; -: Unable to determine.

#FFA: Free fatty acids; CAR: Carnitine.

Table S3. Top 20 differential metabolites among organic acid and its metabolites (Class I)

Compounds	Class II	VIP	P-value	Log2FC	Regulation	Type*
2-Hydroxy-2H-benzo[h]chromene-2-carboxylate	Organic acid and Its derivatives	1.7276	0.0000	7.6985	up	B
DL-3-Phenyllactic acid	Organic acid and Its derivatives	1.4796	0.0027	5.0733	up	A
L-3-Phenyllactic acid	Organic acid and Its derivatives	1.5129	0.0016	4.1981	up	A
Citrate	Organic acid and Its derivatives	1.5079	0.0000	4.0989	up	B
Caftaric acid	Organic acid and Its derivatives	1.5832	0.0000	4.0826	up	A
L-2-Aminoadipic acid	Organic acid and Its derivatives	1.6545	0.0000	3.9689	up	A
5-O-(1-carboxyvinyl)-3-phosphate	Phosphoric acids	1.5244	0.0000	3.7087	up	B
Foscarnet	Phosphoric acids	1.3192	0.0012	3.5580	up	B
Cytidine 3'-monophosphate	Organic acid and Its derivatives	1.5223	0.0000	3.4452	up	B
Uric acid	Organic acid and Its derivatives	1.5168	0.0000	3.4136	up	A
1-Hydroxy-2-naphthoate	Organic acid and Its derivatives	1.5689	0.0000	3.3312	up	A
Colneleic acid	Organic acid and Its derivatives	1.5373	0.0000	3.2672	up	B
1-(2-carboxyphenylamino)-1-deoxy-D-ribulose 5-phosphate	Organic acid and Its derivatives	1.6029	0.0000	3.2555	up	B
Citric Acid	Organic acid and Its derivatives	1.3310	0.0000	3.1951	up	A
Isocitric acid	Organic acid and Its derivatives	1.3310	0.0000	3.1951	up	A
(3R)-3-Hydroxy-2-oxo-4-phosphonooxybutanoate	Organic acid and Its derivatives	1.3762	0.0000	3.1696	up	B
4-Maleylacetoacetic acid	Organic acid and Its derivatives	1.5376	0.0000	3.1318	up	B
Octadec-9-ene-1,18-dioic-acid	Organic acid and Its derivatives	1.5328	0.0000	3.0919	up	B
Glycerol 3-Phosphate	Phosphoric acids	1.3783	0.0000	2.9118	up	A
4-Fumarylacetoacetic acid	Organic acid and Its derivatives	1.5243	0.0000	7.6985	up	B

*A: Endogenous metabolites; B: Exogenous metabolites; -: Unable to determine.

Table S4. Top 20 differential metabolites among nucleotide and its metabolites (Class I)

Compounds	Class II	VIP	P-value	Log2FC	Regulation	Type*
UDP-N-acetyl-3-O-(1-carboxy vinyl)-D-glucosamine	Nucleotide and Its metabolites	1.5253	0.0000	6.3382	up	A
Orotidylic acid	Nucleotide and Its metabolites	1.6617	0.0000	5.9360	up	A
Uridine-5'-diphospho-N-acetyl galactosamine disodium salt	Nucleotide and Its metabolites	1.4903	0.0000	5.8792	up	A
2',3'-Dideoxyinosine	Nucleotide and Its metabolites	1.5107	0.0000	4.1137	up	B
Guanosine-5'-monophosphate	Nucleotide and Its metabolites	1.4209	0.0001	4.0186	up	A
2'-O-Methylguanosine	Nucleotide and Its metabolites	1.5673	0.0000	3.8129	up	-
Xanthosine	Nucleotide and Its metabolites	1.5704	0.0000	3.7756	up	A
Xanthosine-5'-monophosphate	Nucleotide and Its metabolites	1.4647	0.0000	3.7302	up	A
Cytidine 5'-Diphosphocholine	Nucleotide and Its metabolites	1.3195	0.0000	3.6534	up	A
Cytidine-5-Monophosphate	Nucleotide and Its metabolites	1.6299	0.0000	3.4899	up	A
2-(Dimethylamino)Guanosine	Nucleotide and Its metabolites	1.5509	0.0000	3.4728	up	A
2'-Deoxyuridine	Nucleotide and Its metabolites	1.5674	0.0000	3.4399	up	A
2'-O-methyluridine	Nucleotide and Its metabolites	1.4415	0.0000	3.3737	up	-
3-Deoxyadenosine	Nucleotide and Its metabolites	1.5659	0.0000	3.3705	up	-
2'-Deoxyadenosine	Nucleotide and Its metabolites	1.5659	0.0000	3.3705	up	A
dTDP-3-acetamido-3,6-dideoxy-alpha-D-galactopyranose	Nucleotide and Its metabolites	1.4686	0.0000	3.3705	up	A
Adenosine 5'-Monophosphate	Nucleotide and Its metabolites	1.4973	0.0000	3.1438	up	A
3'-Adenylic acid	Nucleotide and Its metabolites	1.3862	0.0000	3.1238	up	A
Deoxyguanosine 5'-monophosphate(dGMP)	Nucleotide and Its metabolites	1.2757	0.0001	3.1215	up	A
2',3'-Cyclic GMP	Nucleotide and Its metabolites	1.5623	0.0000	6.3382	up	A

*A: Endogenous metabolites; B: Exogenous metabolites; -: Unable to determine.

Table S5. Top 20 differential metabolites among benzene and substituted derivatives (Class I)

Compounds	Class II	VIP	P-value	Log2FC	Regulation	Type*
Didox	Benzene and substituted derivatives	1.7489	0.0000	7.1169	up	B
Pyrazolynate	Benzene and substituted derivatives	1.1312	0.0012	6.5278	up	B
H-Lys-Tyr-Lys-OH Acetate salt	Benzene and substituted derivatives	1.1079	0.0030	6.4284	up	B
3-Chloroaniline	Benzene and substituted derivatives	1.6385	0.0000	6.2468	up	B
4-Chloroaniline	Benzene and substituted derivatives	1.6716	0.0000	5.9754	up	B
Ethylparaben	Benzene and substituted derivatives	1.0838	0.0195	5.3480	up	A
Ranolazine	Phenolics	1.2158	0.0029	4.7133	up	B
3-Chlorophenol	Phenolics	1.6750	0.0000	4.6661	up	B
2-(4-hydroxyphenyl) propionate	Phenolic acids	1.5066	0.0012	4.1401	up	B
Fenbufen	Benzene and substituted derivatives	1.4057	0.0006	3.7822	up	B
Resveratrol	Benzene and substituted derivatives	1.5876	0.0000	3.5060	up	B
[(1S,6S,7S,8R,9R,13R,14R,16S,18R)-8-acetyloxy-11-ethyl-5,7,14-trihydroxy-6,16,18-trimethoxy-13-(methoxymethyl)-11-azahexacyclo[7.7.2.12,5.01,10.03,8.013,17]nonadecan-4-yl] benzoate	Benzene and substituted derivatives	1.5845	0.0000	3.4882	up	B
2-Butyl-3-(4-hydroxybenzoyl)benzofuran	Benzene and substituted derivatives	1.5211	0.0000	3.4736	up	B
5-Sulfosalicylic acid	Benzene and substituted derivatives	1.5589	0.0000	3.3866	up	B
Luteoskyrin	Benzene and substituted derivatives	1.5068	0.0000	3.1847	up	B
3,5-Dichlorosalicylic acid	Benzene and substituted derivatives	1.3338	0.0000	2.9977	up	B
4-Hydroxybenzyl alcohol	Benzene and substituted derivatives	1.4763	0.0000	2.9152	up	A
Monoethylglycinexylidide	Benzene and substituted derivatives	1.4229	0.0000	2.8178	up	B
Rubiadin	Benzene and substituted derivatives	1.5401	0.0000	2.8048	up	B
Benzothiazole	Benzene and substituted derivatives	1.4968	0.0000	2.7942	up	B

*A: Endogenous Metabolites; B: Exogenous metabolites; -: Unable to determine.

Table S6. Top 20 differential metabolites among heterocyclic compounds (Class I)

Compounds	Class II	VIP	P-value	Log2FC	Regulation	Type*
Triamterene	Heterocyclic compounds	1.7604	0.0000	12.2284	up	A
Cetylpyridinium	Pyridine and pyridine derivatives	1.2008	0.0325	7.8681	up	B
Gadovist	Heterocyclic compounds	1.3597	0.0022	6.2290	up	B
Apicidin	Heterocyclic compounds	1.1238	0.0030	6.1361	up	B
Aerophobin 1	Heterocyclic compounds	1.0893	0.0013	6.0437	up	B
Psychotridine	Heterocyclic compounds	1.1468	0.0015	5.9814	up	B
2-(3-(1-(((4E,10E)-2,9-Dioxo-12-isopropyl-1,8-diazacyclododecane-4,10-diene-3-yl)carbamoyl)-2-methylpropyl)ureido)-3-methylbutyric acid	Heterocyclic compounds	1.1910	0.0023	5.6905	up	B
Enniatin B	Heterocyclic compounds	1.1363	0.0005	5.3945	up	B
Methotrexate	Pteridines and derivatives	1.5996	0.0000	4.4023	up	A
scytophycin C	Heterocyclic compounds	1.1318	0.0030	4.2053	up	B
11-Hydroxy-THC	Heterocyclic compounds	1.3160	0.0000	3.2764	up	B
Isoduartin methyl Ether	Heterocyclic compounds	1.3860	0.0000	3.2588	up	B
Ditalimfos	Heterocyclic compounds	1.3665	0.0000	3.1504	up	B
N-(3-Chlorophenyl)-6,7-dimethoxyquinazolin-4-amine	Heterocyclic compounds	1.2487	0.0009	3.0038	up	B
Coumarin	Heterocyclic compounds	1.3640	0.0000	2.9970	up	A
Psoralen	Heterocyclic compounds	1.4496	0.0000	2.9099	up	B
Sempervirene	Heterocyclic compounds	1.3492	0.0001	2.9079	up	B
Thiobinupharidine	Heterocyclic compounds	1.3565	0.0000	2.8596	up	B
Levofloxacin	Heterocyclic compounds	1.5917	0.0000	2.8218	up	B
magnesium;3-[(3R,21S,22S)-16-ethenyl-11-ethyl-3-methoxycarbonyl-12,17,21,26-tetramethyl-4-oxo-23,25-diaza-7,24-diazanidahexacyclo[18.2.1.15,8.110,13.115,18.02,6]hexacosa-1,5,8(26),9,13(25),14,16,18,20(23)-nonaen-22-yl]propanoic acid	Heterocyclic compounds	1.4627	0.0000	2.6848	up	B

*A: Endogenous Metabolites; B: Exogenous metabolites; -: Unable to determine.

Table S7. Top 20 differential metabolites among carbohydrates and its metabolites (Class I)

Compounds	Class II	VIP	P-value	Log2FC	Regulation	Type*
1,6-di-O-phosphono-D-fructose	Phosphate sugars	1.4145	0.0000	3.7102	up	A
D-Fructose 6-Phosphate-Disodium Salt	Phosphate sugars	1.4941	0.0000	3.5280	up	A
2-Deoxyribose 1-Phosphate	Phosphate sugars	1.6200	0.0000	3.1916	up	A
L-Fucose	Sugars	1.4542	0.0000	2.6731	up	A
Glucoronic Acid	Sugar acids	1.4526	0.0000	2.3066	up	A
N-Acetylglucosamine 1-Phosphate	Phosphate sugars	1.2356	0.0000	2.2835	up	A
Glucose	Sugars	1.3214	0.0000	1.9653	up	A
Ribulose-5-Phosphate	Phosphate sugars	1.1257	0.0000	1.8081	up	A
Ribose 1-phosphate	Phosphate sugars	1.1257	0.0000	1.8081	up	A
D-Xylulose 5-phosphate	Phosphate sugars	1.1257	0.0000	1.8081	up	A
D-Arabinose 5-Phosphate	Phosphate sugars	1.1257	0.0000	1.8081	up	A
Fructose	Sugars	1.2569	0.0000	1.6928	up	A
Mannose	Sugars	1.2899	0.0000	1.6928	up	A
Talose	Sugars	1.2899	0.0000	1.6928	up	B
Allose	Sugars	1.2899	0.0000	1.6928	up	B
L-Gulose	Carboxylic acids and derivatives	1.2899	0.0000	1.6928	up	A
Aldehydo-D-altrose	Sugars	1.2899	0.0000	1.6928	up	B
Sedoheptulose 7-phosphate	Phosphate sugars	1.0958	0.0001	1.5650	up	A
Ribitol	Sugar Alcoholss	1.0836	0.0001	1.4765	up	B
Arabitol	Sugar Alcoholss	1.0279	0.0002	1.4010	up	A

*A: Endogenous Metabolites; B: Exogenous metabolites; -: Unable to determine.

Table S8. Top 20 differential metabolites among GP (Class I)

Compounds	Class II #	VIP	P-value	Log2FC	Regulation	Type*
methylcarbamyl PAF	PC	1.0993	0.0014	76.03	6.2484	A
1-Oleoyl lysophosphatidic acid sodium salt	PA	1.5540	0.0121	73.32	6.1962	A
5'-Deoxyadenosine	PA	1.5277	0.0000	17.49	4.1283	A
beta-Glycerophosphoric acid	GP	1.3881	0.0000	7.82	2.9675	A
LPC(20:5/0:0)	LPC	1.3603	0.0000	5.91	2.5624	A
LPC(0:0/20:5)	LPC	1.3603	0.0000	5.91	2.5624	A
O-Phosphorylethanolamine	Others	1.4258	0.0000	5.20	2.3779	A
LPC(20:3/0:0)	LPC	1.1572	0.0000	4.75	2.2494	A
LPC(22:6/0:0)	LPC	1.2825	0.0000	4.26	2.0906	A
LPC(0:0/22:6)	LPC	1.2825	0.0000	4.26	2.0906	A
LPC(0:0/20:4)	LPC	1.1578	0.0000	3.90	1.9622	A
LPC(20:4/0:0)	LPC	1.1578	0.0000	3.90	1.9622	A
LPC(0:0/22:5)	LPC	1.1961	0.0000	3.79	1.9231	A
LPC(22:5/0:0)	LPC	1.1961	0.0000	3.79	1.9231	A
LPC(0:0/18:1)	LPC	1.1356	0.0000	3.67	1.8739	A
LPC(18:1/0:0)	LPC	1.0678	0.0000	3.29	1.7187	A
1-(9Z-octadecenoyl)-sn-glycero-3-phosphocholine	LPC	1.0600	0.0000	3.22	1.6878	A
LPC(0:0/20:3)	LPC	1.0979	0.0001	3.14	1.6526	A
LPC(O-16:0/2:0)	LPC	1.0799	0.0000	2.97	1.5719	A
LPC(0:0/14:0)	LPC	1.0341	0.0000	2.79	1.4824	A

*A: Endogenous Metabolites; B: Exogenous metabolites; -: Unable to determine.

#PC: Phosphatidylcholine; PA: Phosphatidic acid; GP: Glycerophospholipids; LPC: Lysophosphatidylcholine.

Table S9. Top 20 differential metabolites among alcohol and amines (Class I)

Compounds	Class II	VIP	P-value	Log2FC	Regulation	Type*
Proguanil	Amines	1.76	0.0000	11.1915	up	B
Palmitoylethanolamide	Amines	1.53	0.0000	4.7405	up	A
N-(2-hydroxyethyl)stearamide	Amines	1.53	0.0000	4.5876	up	A
2-(α-D-mannosyl)-3-phosphate glyceride	Amines	1.31	0.0002	3.9270	up	A
Palmitoylethanolamide（PEA）	Amines	1.44	0.0000	3.8402	up	A
[(2R,3S,4R,5R)-5-[5-amino-4-[(4-amino-4-oxo-butanoyl)amino]imidazol-1-yl]-3,4-dihydroxy-tetrahydrofuran-2-yl]methyl dihydrogen phosphate	Amines	1.37	0.0004	3.5535	up	B
D-myo-Inositol-4-phosphate (ammonium salt)	Alcohols	1.46	0.0000	3.2467	up	A
beta-2,3,5,6-Tetrachloro-1,4-cyclohexanediol	Alcohols	1.38	0.0000	3.0181	up	B
Methazolamide	Amines	1.34	0.0000	2.9700	up	B
Crotetamide	Amines	1.33	0.0080	2.8002	up	B
Triethanolamine	Alcohols	1.21	0.0009	2.7633	up	B
Linoleylethanolamide	Amines	1.29	0.0000	2.7282	up	A
Schradan	Amines	1.43	0.0000	2.4170	up	B
Ethyl(E,4S)-4-[[3-hydroxy-2-[[(2S)-2-[(3-hydroxy-2-methylbenzoyl)amino]-3-methylbutanoyl]amino]propanoyl]amino]-6-methylhept-2-enoate	Amines	1.22	0.0000	2.0773	up	B
Spermidine	Polyamines	1.43	0.0000	1.9977	up	A
Aniline	Amines	1.49	0.0000	1.8055	up	A
Putrescine	Polyamines	1.08	0.0000	1.6871	up	A
Guanidine	Polyamines	1.09	0.0023	1.5432	up	A
Bis(1-inositol)-3,1'-phosphate 1-phosphate	Alcohols	1.32	0.0000	1.4635	up	B
Biotinamide	Polyamines	1.07	0.0012	1.0834	up	A

*A: Endogenous Metabolites; B: Exogenous metabolites; -: Unable to determine.

Table S10. Differential metabolites among aldehyde, ketones, esters (Class I)

Compounds	Class II	VIP	P-value	Log2FC	Regulation	Type*
D-Arabinono-1,4-lactone	Esters	1.5624	0.0000	4.0571	up	B
Hydron;2-hydroxypropane-1,2,3-tricarboxylate	Esters	1.5108	0.0000	3.3339	up	B
(1-Heptan-2-yl-2-methylindol-3-yl)-naphthalen-1-ylmethanone	Ketones	1.3579	0.0000	3.0923	up	B
Moniliformin	Ketones	1.5320	0.0000	2.9339	up	B
Diethyl hydrogen phosphate	Esters	1.1276	0.0006	2.7875	up	A
2-Tetradecanone	Ketones	1.2671	0.0004	2.6451	up	B
Fumagillin	Esters	1.5152	0.0000	2.6317	up	B
2',4-Dihydroxy-4',6'-dimethoxychalcone	Ketones	1.2022	0.0000	2.5732	up	B
5-Quinoxalin-6-ylmethylene-thiazolidine-2,4-dione	Ketones	1.3866	0.0000	2.3197	up	B
Methyl dihydrogen phosphate	Esters	1.3964	0.0000	2.0368	up	A
xi-2,3-Dihydro-3,5-dihydroxy-6-methyl-4H-pyran-4-one	Ketones	1.5704	0.0000	1.9578	up	B
D-alpha-glutamyl phosphate	Esters	1.3279	0.0000	1.6413	up	B
Di-n-propyl-phthalate	Esters	1.1219	0.0000	1.5436	up	A
Risperidone	Ketones	1.0864	0.0000	1.4121	up	B
Methyl-2-pyrrolidone-5-carboxylate	Esters	1.0537	0.0000	1.1446	up	A
2,6-Dioxo-6-phenylhexa-3-enoate	Esters	1.0796	0.0000	0.9632	up	B
Chloroacetyl chloride	Ketones	1.4759	0.0000	-0.4236	down	B
Tetrahydrobisdemethoxydiferuloylmethane	Ketones	1.3464	0.0000	-1.0156	down	B

*A: Endogenous Metabolites; B: Exogenous metabolites; -: Unable to determine.

Table S11. Top 20 differential metabolites among Others

Compounds	Class I	Class II	VIP	P-value	Log2FC	Regulation	Type*
4'-O-Methylnorbelladine	Alkaloids	Alkaloids	1.7379	0.0000	10.3234	up	B
Sequinavir	Others	Medicine	1.1345	0.0050	7.2004	up	B
Narirutin	Flavonoids	Flavonoid	1.7504	0.0000	6.6873	up	B
Prostaglandin F2-biotin	Hormones and hormone related compounds	Hormones and hormone related compounds	1.0699	0.0007	5.2840	up	B
2,22-Dideoxy-3-dehydro ecdysone	Hormones and hormone related compounds	Hormones and hormone related compounds	1.6315	0.0000	5.1311	up	A
Eriodictyol	Flavonoids	Dihydroflavone	1.5248	0.0003	5.0541	up	B
Ginsenoside F3	Steroids	Steroidal saponins	1.0246	0.0003	4.8319	up	B
Biochanin A	Flavonoids	Isoflavones	1.5744	0.0000	4.6945	up	B
13,14-Dihydro PGF-1a	Hormones and hormone related compounds	Hormones and hormone related compounds	1.4890	0.0000	4.2878	up	B
4-Hydroxytryptamine	Tryptamines,Cholines,Pigments	Tryptamines	1.0923	0.0004	4.2151	up	A
Vitamin K	CoEnzyme and vitamins	CoEnzyme and vitamins	1.5121	0.0000	4.0583	up	B
13(S)-HODE cholesteryl ester	Others	Others	1.6323	0.0000	3.6811	up	-
25-Hydroxyvitamin D2	CoEnzyme and vitamins	CoEnzyme and vitamins	1.4182	0.0000	3.3984	up	A
Atipamezole hydrochloride	Others	Medicine	1.3163	0.0001	2.9232	up	B
Axitinib	Others	Others	1.0145	0.0001	2.8373	up	-
Eicosapentaenoyl Serotonin	Tryptamines,Cholines,Pigments	Tryptamines	1.5194	0.0000	2.7685	up	B
Pyridoxine	CoEnzyme and vitamins	CoEnzyme and vitamins	1.0897	0.0278	2.7662	up	A
2-Hydroxy-2,3-dihydrogenistein	Flavonoids	Isoflavones	1.5220	0.0000	2.6264	up	B
Dimethyldisulfide	Others	Others	1.5622	0.0000	2.6020	up	B
N-(Cyclopropylmethyl)-7-[3,5-dihydroxy-2-(3-hydroxy-5-phenylpent-1-EN-1-YL)cyclopentyl]hept-5-enamide	Hormones and hormone related compounds	Hormones and hormone related compounds	1.2603	0.0000	2.5601	up	B

*A: Endogenous Metabolites; B: Exogenous metabolites; -: Unable to determine.

Table S12. Top 20 differential metabolites among small peptide (Class II)

Compounds	VIP	P-value	Log2FC	Regulation	Type*
Glu-Asp-Thr-Glu	1.7468	0.0000	8.5061	up	A
Ser-Lys-Phe-Leu-Lys	1.7520	0.0000	8.1784	up	A
Tyr-Leu-Ala-Lys	1.2742	0.0115	8.0426	up	A
Pro-Ala-Leu-Phe-Leu	1.1993	0.0044	7.0224	up	A
Lys-His-Phe-Arg	1.1827	0.0041	6.9255	up	A
Phe-Ala-Arg-Gln-Lys	1.0997	0.0033	6.5654	up	A
Leu-Val-Leu-Gly-Phe	1.2098	0.0051	6.5399	up	A
Arg-Leu-Val-Glu	1.1873	0.0050	6.4414	up	A
Lys-His-Ala-Val-Ser	1.0380	0.0013	6.0555	up	A
Gly-Leu-Arg-Val-Phe	1.1591	0.0009	6.0509	up	A
Gly-Lys-Lys-Gln-Leu	1.2052	0.0016	5.9590	up	A
Ile-Gly-Lys-Ile-Phe	1.0313	0.0011	5.9303	up	A
Leu-Arg-Pro-Thr-Leu	1.1219	0.0011	5.9261	up	A
Thr-Lys-Gln-Lys	1.1239	0.0006	5.9106	up	A
Ser-Val-Lys-Arg	1.1947	0.0037	5.8303	up	A
Trp-His-Tyr	1.7188	0.0000	5.6956	up	A
Leu-Leu-Val-Val-Tyr	1.1088	0.0014	5.5906	up	A
His-Ile-Lys-Arg	1.0699	0.0001	5.3484	up	A
Ser-Arg-Ile-Lys	1.0738	0.0058	5.2488	up	A
Arg-Lys-His-Arg	1.0328	0.0002	5.0031	up	A

*A: Endogenous Metabolites; B: Exogenous metabolites; -: Unable to determine.

Table S13. Overlaps of differential metabolites between PASI-Cluster 5 and BSA-Cluster 1

Compounds	Class I	Class II	PASI-Pvalue*	BSA-P value#	VIP	Log2FC
Putrescine	Alcohol and amines	Polyamines	0.0679	0.2768	1.0784	1.6871
L-Glutamic Acid	Amino acid and Its metabolites	Amino acids	0.0002	0.0029	1.4128	2.0881
L-Tyrosine	Amino acid and Its metabolites	Amino acids	0.0035	0.0093	1.0282	1.0391
L-Arginine	Amino acid and Its metabolites	Amino acids	0.0044	0.0138	1.1393	1.1377
N-Acetylthreonine	Amino acid and Its metabolites	Amino acid derivatives	0.0152	0.0490	1.4423	2.0227
N-Acetylornithine	Amino acid and Its metabolites	Amino acid derivatives	0.0044	0.0138	1.1393	1.1377
(R)-(-)-2-Phenylglycinol	Amino acid and Its metabolites	Amino acid derivatives	0.0029	0.0433	1.1061	1.0094
Pro-Glu	Amino acid and Its metabolites	Small Peptide	0.0159	0.0341	1.2711	1.3573
Ser-Pro	Amino acid and Its metabolites	Small Peptide	0.0130	0.0239	1.2207	1.6141
N-Isovaleroylglycine	Amino acid and Its metabolites	Amino acid derivatives	0.0036	0.0097	1.0004	1.0090
DL-Arginine	Amino acid and Its metabolites	Amino acids	0.0018	0.0048	1.0523	1.1088
N-(2-Furoyl)glycine	Amino acid and Its metabolites	Amino acid derivatives	0.0002	0.0014	1.3257	1.4349
1-Hydroxylamino-2-phenylethane	Benzene and substituted derivatives	Benzene and substituted derivatives	0.0029	0.0433	1.1061	1.0094
Benzaldehyde	Benzene and substituted derivatives	Benzene and substituted derivatives	0.0062	0.0346	1.0243	0.8649
2,5-Dihydroxybenzoic acid	Benzene and substituted derivatives	Benzene and substituted derivatives	0.2082	0.3291	1.5176	2.0450
Neburon	Benzene and substituted derivatives	Benzene and substituted derivatives	0.0011	0.0009	1.3530	1.3198
Benzydamine	Benzene and substituted derivatives	Benzene and substituted derivatives	0.0001	0.0008	1.1615	1.7541
3-(4-Hydroxyphenyl)chroman-7-ol	Benzene and substituted	Benzene and substituted	0.0049	0.0065	1.1843	1.4077

	derivatives	derivatives				
3-Methoxy-4',5-dihydroxy-trans-stilbene	Benzene and substituted derivatives	Benzene and substituted derivatives	0.0044	0.0065	1.1780	1.4147
Arginine Hydrochloride	Others	Medicine	0.0030	0.0058	1.0881	1.0936
Purine	Nucleotide and Its metabolites	Nucleotide and Its metabolites	0.0097	0.0314	1.0502	0.8737
Pyrrolidine	Heterocyclic compounds	Heterocyclic compounds	0.0072	0.0142	1.2279	1.1897
Firefly luciferin	Heterocyclic compounds	Heterocyclic compounds	0.0059	0.0023	1.4657	0.4679
N-(2-hydroxyethyl)-3-pyridinecarboxamide	Heterocyclic compounds	Heterocyclic compounds	0.0193	0.0269	1.0690	0.9924
Tropate	Organic acid and Its derivatives	Organic acid and Its derivatives	0.0004	0.0006	1.0872	0.9884
D-Quinovose	Carbohydrates and Its metabolites	Carbohydrates and Its metabolites	0.0285	0.0683	1.0949	1.1895
Citrate	Organic acid and Its derivatives	Organic acid and Its derivatives	0.0111	0.0366	1.5079	4.0989

*PASI-P value: P-value of spearman correlation between differential metabolite and PASI score;

#BSA-P value: P-value of spearman correlation between differential metabolites and BSA score.
